# METTL3 Modulates Radiation‐Induced Cardiac Fibrosis via the Akt/mTOR Pathway

**DOI:** 10.1096/fj.202403143RRRR

**Published:** 2025-06-05

**Authors:** Shunsong Qiao, Chao Tang, Jingjing Zhu, Yu Feng, Li Xiang, Jing Zhu, Xiaosong Gu

**Affiliations:** ^1^ Department of Cardiology The Second Affiliated Hospital of Soochow University Suzhou China; ^2^ Department of Endocrinology The Second Affiliated Hospital of Soochow University Suzhou China; ^3^ Department of Rheumatology and Immunology The Fourth Affiliated Hospital of Soochow University Suzhou China

**Keywords:** Akt signaling, heart failure, METTL3, myocardial fibrosis, radiation

## Abstract

While thoracic radiotherapy represents a mainstay therapeutic modality for malignancies, its cardiotoxic sequelae, particularly radiation‐induced cardiac fibrosis (RICF), significantly limit clinical outcomes. Emerging evidence implicates the N6‐methyladenosine (m6A) methyltransferase METTL3 in pathological cardiac remodeling, though its mechanistic involvement in radiation‐associated fibrogenesis remains enigmatic. This investigation elucidates the epigenetic regulation of METTL3 in RICF pathogenesis. X‐ray‐modulated RICF mice models were constructed to investigate the role of METTL3 in cardiac fibroblasts. Simultaneously, METTL3 overexpression and silencing were conducted on fibroblasts and mice hearts to evaluate pro‐fibrotic protein expression, cardiac fibrosis, and heart function. Radiation impairs cardiac function and induces myocardial fibrosis. Elevated METTL3 expression was observed in irradiated mouse hearts and cardiac fibroblasts. During irradiation, METTL3 promoted fibroblast proliferation and differentiation into myofibroblasts. Overexpression of METTL3 in cardiac fibroblasts was associated with increased expression of pro‐fibrotic proteins and intensified fibrosis, and silencing of METTL3 attenuated these adverse effects. Mechanistically, METTL3 promotes m6A modification of Akt mRNA and enhances its stability by recognizing the m6A‐reading protein IGF2BP1, which activates the Akt/mTOR signaling pathway to promote fibroblast proliferation and differentiation toward myofibroblasts, thereby inducing cardiac fibrosis. Furthermore, pharmacological administration of STM2457, a highly selective METTL3 inhibitor, effectively ameliorated cardiac fibrosis in mice. Our findings establish METTL3 as a novel epigenetic regulator of RICF through m6A‐Akt/mTOR axis activation. The demonstrated efficacy of METTL3‐targeted intervention provides mechanistic justification for developing precision cardioprotective strategies during radiotherapy. This work advances our understanding of epitranscriptomic control in radiation‐associated cardiotoxicity and highlights the translational potential of m6A‐targeted therapies.

## Graphical abstract

This study demonstrates that X‐ray radiation induces upregulation of METTL3 in mouse hearts, which is associated with reduced cardiac function and exacerbated cardiac fibrosis. X‐irradiation significantly elevates α‐SMA, Collagen I, and TGF‐β1 levels, with METTL3 knockdown mitigating this effect and attenuating X‐irradiation‐induced fibrosis. METTL3 enhances the stability of Akt mRNA by recognizing the m6A reader protein IGF2BP1, thereby promoting the phosphorylation of Akt, which facilitates the proliferation, migration, and fibrosis of cardiac fibroblasts.

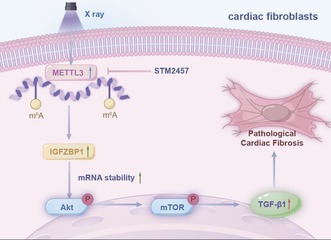

**Abbreviations**
CFs: cardiac fibroblasts
ECM: extracellular matrix
IR: ionizing radiation
m6A: N6‐methyladenosine modification
METTL3: methyltransferase‐like 3
RIHD: radiation‐induced heart disease
SR: Sirius red
TGF‐β1: transforming growth factor β1
WGA: wheat germ agglutinin
α‐SMA: smooth muscle α‐actin

## 1 Introduction

Radiotherapy remains a cornerstone in oncologic management, yet its cardiovascular complications pose substantial limitations to long‐term survivorship. Accumulating clinical evidence has established a dose‐dependent causal relationship between mediastinal radiotherapy and cardiovascular morbidity [1]. Radiation‐induced heart disease (RIHD) encompasses a heterogeneous spectrum of cardiac pathologies, ranging from pericarditis and coronary artery disease to myocardial fibrosis and congestive heart failure [2, 3]. Epidemiological analyses reveal a linear no‐threshold risk association, with 14% of patients developing RIHD following 1 Gy cardiac exposure [4]. Notably, dose‐escalation studies demonstrate a 7.4% annual increase in major adverse cardiac events among breast cancer survivors, typically manifesting as delayed complications (5–20 years post‐treatment) [1]. These compelling clinical observations underscore the imperative to decipher the molecular underpinnings of radiation cardiotoxicity.

Within this pathophysiological continuum, cardiac fibroblasts (CFs) serve as central mediators of fibrotic remodeling through dynamic regulation of extracellular matrix (ECM) homeostasis [5]. Ionizing radiation triggers maladaptive phenotypic switching in CFs, orchestrated by multilayered activation of pro‐fibrotic signaling networks across transcriptomic, post‐transcriptomic, and proteomic dimensions [6]. This radiation‐induced transformation into matrix‐synthetic myofibroblasts culminates in disproportionate collagen deposition and architectural distortion of myocardial tissue, thereby establishing cardiac fibrosis as a hallmark pathological feature of RIHD [7]. Elucidating the regulatory mechanisms governing fibroblast activation thus represents a critical knowledge gap in radiation cardiotoxicity research.

Emerging insights implicate N6‐methyladenosine (m6A) epitranscriptomic regulation in cardiovascular pathophysiology, particularly through METTL3 (methyltransferase‐like 3)‐mediated methylation that governs RNA metabolism and translational efficiency [8, 9]. Accumulating preclinical evidence positions METTL3 as a pleiotropic modulator of myocardial stress responses, influencing pressure overload adaptation, ischemia–reperfusion injury, and post‐infarction remodeling [10]. Notably, the interface between m6A modification and DNA damage response pathways assumes particular relevance given the primacy of radiation‐induced DNA lesions in RIHD pathogenesis [4, 11, 12]. Despite these advances, the specific involvement of METTL3 in radiation‐associated fibrotic remodeling remains undefined, creating a critical conceptual void in our understanding of epitranscriptomic control in RIHD.

This investigation systematically examines the mechanoregulatory role of METTL3 in radiation‐driven cardiac fibrosis. Using a preclinical murine model of RIHD, we elucidate the functional consequences of METTL3 dysregulation on cardiac fibroblast activation and ECM dysregulation. Through integrated molecular interrogation, we further unravel the m6A‐dependent mechanism underlying Akt/mTOR pathway hyperactivation, thereby establishing a novel epitranscriptomic axis in radiation cardiotoxicity.

## 2 Materials and Methods

### 2.1 Animals Models

All animal experimental procedures adhered to the guidelines established by the Animal Care and Use Committee and the Ethics Committee of the Second Affiliated Hospital of Soochow University (No. 2023076). C57BL/6 mice (SPF) were purchased from Suzhou Specific Bio‐technology Co. All experiments were performed according to ARRIVE guidelines. The mice were irradiated after a 1‐week acclimatization period, euthanized by carbon dioxide asphyxiation 4 weeks later, and heart samples were collected.

### 2.2 Primary Cardiac Fibroblasts

Cardiac fibroblasts were isolated from postnatal day 1–3 C57BL/6 mice. Hearts were rapidly excised and immersed in DMEM medium (10‐013‐CV, Corning, USA) pre‐cooled, well sheared (< 1 mm^3^) and digested in 0.25% trypsin (Thermo Fisher, USA) and 0.1% collagenase II (BioFroxx, Germany) at 37°C. Following digestion, cells were collected, centrifuged at 1000 rpm for 3 min, then cultured in DMEM supplemented with 10% fetal bovine serum (Thermo Fisher, USA), 100 U/mL penicillin, and 100 μg/mL streptomycin (Thermo Fisher, USA) for 2 h. The medium was then changed to remove weakly adhered cells. Isolated purified fibroblasts were further incubated in DMEM at 37°C in humidified air with 5% CO_2_ and 95% O_2_. Routine passages were performed every 2–3 days. The cardiac fibroblasts were then passaged and cultured, and the purity of CF was detected with flow cytometry (Figure S12A). P1‐2 was used for experiments.

### 2.3 Flow Cytometry

Extracted cardiac fibroblasts were resuspended in PBS containing 2% FBS, fixed with pre‐cooled 4% paraformaldehyde for 15 min, and incubated for 1 h at 4°C away from light by adding 0.1% Triton X‐100 permeabilization treatment for 20 min, followed by the addition of FSP1 antibody. Wash and resuspend in the flow buffer. Detection was performed using a multi‐laser flow cytometer (BD FACSVerse) configured with a channel‐compatible fluorescent dye (FITC). Compensatory adjustments were made to correct for fluorescence signal overlap with single‐stained samples. Analysis and graphing were performed using FlowJo software.

### 2.4 Radiation Exposure

Mice were randomly assigned to one of three groups: control group, 10 Gy irradiation group, and 20 Gy irradiation group. Subsequently, mice underwent a single irradiation session and were fed for 28 days. Irradiation was conducted using a single‐field technique focused on the chest. The irradiation field had dimensions of 1 × 1 cm^2^ and a depth of 1.5 cm. The source was located 100 cm from the skin. The irradiation volume was either 10 Gy or 20 Gy and was delivered using a linear accelerator (Elekta Synergy, UK) with a dose rate of 600 cGy/min. Radiation energy was 6 MV X‐ray [13]. Hearts were collected for histologic analysis 4 weeks after single‐dose irradiation.

Cardiac fibroblasts were divided into five groups and irradiated with 0, 2, 5, 10, and 20 Gy X‐rays. Cells were irradiated by the same accelerator and dose rate, but with a beam energy of 4 MV X‐rays. Primary cardiac fibroblasts were seeded on culture plates overnight before exposure to incremental doses of ionizing radiation (0, 2, 5, 10, and 20 Gy) [14].

### 2.5 Bioinformatics Studies

The post‐radiation cardiac injury RNA‐seq dataset was retrieved from the GEO database (https://www.ncbi.nlm.nih.gov/geo/), and the GSE218447 dataset was selected and downloaded. Pathway enrichment analysis was performed using the “cluster.” Pathways with a *p*‐value < 0.05 were considered significantly enriched. Selected significant pathways were plotted as bar graphs.

### 2.6 m6A Dot Blot Assay

Mouse hearts and CFs RNA were extracted using TRIzol reagent (Thermo Fisher Scientific, USA) following the manufacturer's instructions. RNA concentration was measured using a NanoDrop ND‐1000 spectrophotometer (Agilent). m6A dot blotting experiments are shown in the “Results” section. Briefly, RNA samples were loaded onto Amersham HybondN+ membranes (Biyun Tian, FFN13) and UV crosslinked. The membranes were blocked with 5% skim milk for 1 h, incubated with m6A antibody (Epigentek, P‐9005‐48, 1:5000) overnight at 4°C, then left with horseradish peroxidase (HRP)‐coupled secondary antibody (Santa Cruz Biotechnology; Sc‐2030) for 1 h at room temperature. Membranes were visualized using an enhanced chemiluminescence detection system (Amersham Biosciences, Piscataway, NJ). The relative signal density at each point was quantified using Image J software.

### 2.7 Quantification of m6A Modifications

Total RNA was enriched with TRIzol (#15596018, Invitrogen) prior to treatment with Deoxyribonuclease I (#04716728001, Sigma‐Aldrich). RNA quality was assessed using NanoDrop. Changes in overall m6A methylation in mRNA were determined using the EpiQuik m6A RNA Methylation Quantification Kit (EPT‐P‐9005‐48, Epigentek). Briefly, 200 ng of RNA was added to assay wells, and capture and detection antibody solutions were applied at appropriate dilutions. The level of m6A methylation is assessed by measuring absorbance at 450 nm and calculated from a standard curve.

### 2.8 Methylated RNA Immunoprecipitation (MeRIP)‐qPCR

RNA samples were fragmented by fragmentation reagent (Invitrogen, AM8740) and incubated with protein G dynabeads‐conjugated m6A antibody (SYSY, 202003) overnight at 4°C. Methylated RNAs were collected by elution buffer (6 mM m6A sodium salt, 50 mM Tris·HCl, 150 mM NaCl, 1 mM EDTA, 1% Nonidet P‐40, 1 U/μL RNase inhibitor); then RNAs were isolated by RNeasy kit (Qiagen). Quantification of m6A‐methylated RNA was performed using the One‐Step Real‐Time RT‐PCR Kit (Invitrogen, 12574018).

### 2.9 Cell Viability Assay

Cell viability was evaluated in a 96‐well plate using Cell Counting Kit‐8 (Cat No. C0039, Beyotime). Cells were plated at 1 × 10^4^ in each well. Absorbance was determined with a Multi‐Plate Reader (Biotek Synergy) at 450 nm prior to the determination of the percentage of cell viability.

### 2.10 Cardiac METTL3 Knockdown and Overexpression

A cardiac METTL3 knockout mouse model was established by injecting lentivirus into the left ventricular cavity, and each mouse was injected with 1 × 10^9^ TU knockout METTL3 virus and negative control virus, respectively. One week later, both groups of mice and the non‐viral transduced group were irradiated at the same time, and the effect was assessed after 4 weeks. METTL3‐specific shRNA (shMETTL3) and scramble shRNA (sh‐scr) were synthesized by OBiO Technology (Shanghai, China). Transduction with shMETTL3 resulted in decreased METTL3 expression, and gene silencing was achieved by transduction of a pre‐designed shRNA duplex designed and synthesized by OBiO Technology (Shanghai, China). CFs were seeded in six‐well plates at 50%–60% confluency and treated with METTL3 shRNA lentiviral particles. After 24 h, cells were cultured in a complete culture medium containing puromycin (1.0 μg/mL) for 12–14 days. Western blot confirmed over 95% METTL3 knockdown in stable cell lines. The target sequence of the knockdown virus was 5′‐GCACCCGCAAGATTGAGTTAT‐3′ (sh‐METTL3), and that of the overexpression virus was 5′‐CGCAAATGGGGCGGTAGGCGTG‐3′ (OE‐METTL3).

### 2.11 Mouse Echocardiography

Mice were anesthetized with 3% isoflurane during induction and 2% isoflurane during maintenance and were placed on a carrier table heated to 37°C. Images were acquired using a VERMON probe (23 MHz) on a VINNO6 Dimension color Doppler ultrasound machine. Analysis was performed using an EchoPac workstation and 2D strain imaging software. High‐quality images were acquired by shaving the precordial region and capturing both short‐axis and apical four‐chamber views of the left ventricle. M‐mode ultrasound was used to assess the LV end‐diastolic internal diameter (LVIDd), end‐systolic internal diameter (LVIDs), ejection fraction (LVEF), and left ventricular shortening rate (LVFS).

### 2.12 Wheat Germ Agglutinin (WGA) Staining

After dewaxing and rehydration, heart slides were incubated with Alexa Fluor 488 (Invitrogen, W11261, 5 μg/mL) coupled to WGA for 10 min at room temperature. Cardiomyocyte area was quantified by capturing images from five hearts per group, with at least 100 cells per heart, using a laser‐scanning confocal microscope (LSM 700, Zeiss). Cell size was quantified using ImageJ software.

### 2.13 Tissue Histological Analysis

Hearts were collected 4 weeks after irradiation, fixed in 4% paraformaldehyde overnight, embedded in paraffin, and sliced into 5 μm‐thick sections starting at the apex. Heart paraffin sections were baked in an oven at 60°C for 60 min, then deparaffinized with xylene. After hydration in graded alcohol and water, sections were stained with a Masson Trichrome kit (Solarbio, Beijing, China).

In these sections, fibrotic tissue was blue and myocardial tissue was red. Heart sections were then stained with a Sirius red kit (Solarbio, Beijing, China), with fibrotic tissue showing red and myocardial tissue yellow. Collagen area was measured using Image‐Pro Plus 6.0.

Hearts were embedded in paraffin, sectioned into 5 μm slices, deparaffinized, rehydrated, and antigen retrieved. Sections were permeabilized with 0.5% Triton X‐100/phosphate buffer saline, then blocked with 5% goat serum (Jackson ImmunoResearch Laboratories, USA) for 1 h at room temperature and incubated with primary antibodies overnight at 4°C. After PBS washing, slices were incubated with the corresponding fluorescence‐coupled secondary antibody for 1 h at room temperature, followed by DAPI (Sigma) counterstaining. Primary antibodies used were α‐SMA (Abcam, ab7817, 1:100) and Vimentin (Proteintech, 60330‐1‐lg, 1:100). Secondary antibodies: goat anti‐mouse Alexa Fluor 488 (Thermo Fisher, A‐11001, 1:500); goat anti‐mouse Alexa Fluor Plus 594 (Thermo Fisher, A32742, 1:500).

### 2.14 Cell Proliferation and Migration Assays

CFs cells were seeded into six‐well plates (2 × 10^5^ cells per well) and cultured for 24 h. Cell proliferation was quantitatively measured using the EdU Apollo‐567 assay kit (RiboBio, Guangzhou, China). Cell nuclei were stained with EdU and 4,6‐diamino‐2‐phenylindole (DAPI) and observed under a fluorescence microscope (Leica, DM 4000, Germany). Cells in each field of view were then counted and analyzed. The nuclear EdU ratio, % with DAPI, was calculated for at least 1000 cells in five randomized views per treatment.

CF cells (40 000 cells per chamber in serum‐free medium) were added to the upper transwell chambers (8 μm wells, Corning, New York, NY, USA), and the lower chambers were filled with 10% fetal bovine serum (FBS) complete medium. After 24 h, CF cells migrating to the lower chambers were fixed, stained, and counted (cataway, NJ).

### 2.15 Western Blot Analysis

Protein extraction from mouse heart tissue and cardiac fibroblasts. Aliquots of 30 μg of protein from each sample were separated by 10% SDS‐polyacrylamide gel electrophoresis (processed as shown in the legend) and transferred to polyvinylidene difluoride membranes (Millipore, Bedford, MA). Membranes were then blocked with 10% skim milk powder for 1 h, incubated with the specific antibody overnight at 4°C overnight, and the secondary antibody (HRP‐coupled anti‐rabbit or anti‐mouse IgG, at an appropriate dilution) for 45 min to 1 h at room temperature. Antibody binding was detected using an enhanced chemiluminescence detection system (Amersham Biosciences, Piscataway, NJ). The primary antibodies used were: anti‐Col I (Abcam, ab21286), anti‐METTL3 (Cell Signaling Technology, 86132S), anti‐GAPDH (Proteintech, 10028230), anti‐TGF‐β1 (Proteintech, 21898‐1‐AP), Phospho‐Akt (CST, #13038), Akt (SantaCruzBiotechnology, #E2121), Phospho‐PI3K (CST, #17366), PI3K (CST, #4249), Phospho‐mTOR (CST, #5536), mTOR (CST, #2983), Phospho‐S6 (CST, #34475), and S6 (SantaCruzBiotechnology, #B2023). Membranes were examined with an Odyssey infrared scanning system (LI‐COR Biosciences, Lincoln, Nebraska, USA), and the western blot images were captured with the band densities quantified using Odyssey 3.0 software.

### 2.16 Statistical Analysis

Results are presented as mean ± SEM and analyzed using one‐way ANOVA with Tukey's test for individual group comparisons. A *p*‐value < 0.05 was considered statistically significant. Analysis and presentation were performed using Prism 9 (GraphPad, USA). The in vitro experiments were repeated at least three times, and consistent results were obtained.

## 3 Results

### 3.1 Radiation Impairs Heart Function and Induces Myocardial Fibrosis Remodeling

**FIGURE 1:**
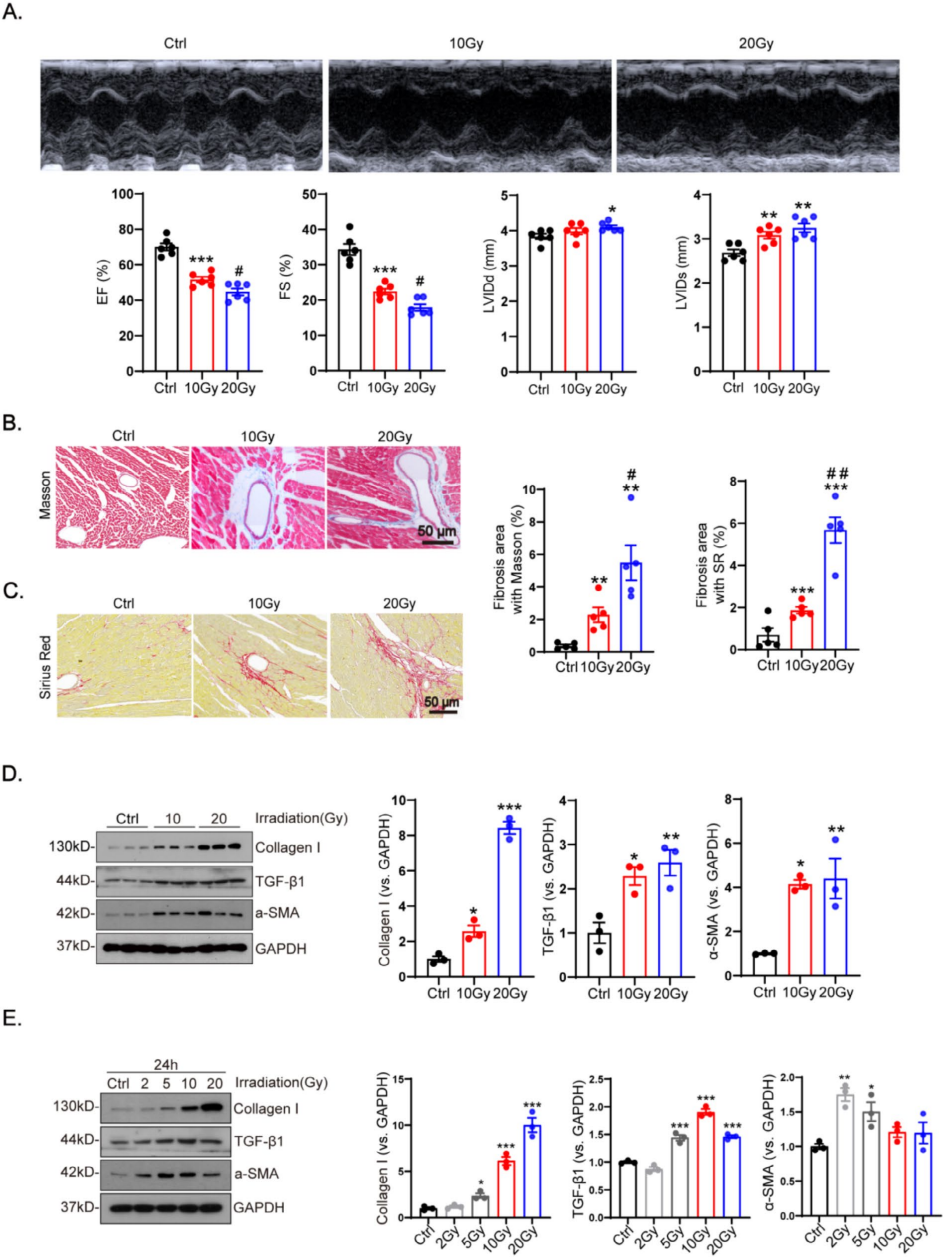
Adult mice and cardiac fibroblasts were subjected to irradiation at doses of 0 (Ctrl), 2, 5, 10, and 20 Gy, with exposure for 4 weeks and 24 h. (A) Heart function parameters included left ventricular ejection fraction (LVEF), left ventricular fractional shortening (LVFS), left ventricular internal diameter (LVID) and left ventricular posterior wall thickness (LVPWd) and were assessed using echocardiography(*n* = 6). (B, C) Tissue fibrosis was evaluated using Masson and Sirius red staining (*n* = 5). Fibrotic markers including α‐SMA, collagen I and TGF‐β1 in heart tissues (D) and cardiac fibroblasts (E) were quantified using western blot (*n* = 3). Data are expressed as mean ± SEM. All results were analyzed using one‐way ANOVA with Tukey's post hoc test and confirmed by three independent experiments. ***p < 0.001, **p < 0.01, *p < 0.05 versus Ctrl; ^##^ *p* < 0.01, ^#^ *p* < 0.05 versus 10 Gy.

The heart function parameters indicated that radiation led to contractile dysfunction. Compared with the controls, left ventricular ejection fraction (LVEF) decreased in mice after irradiation for 4 weeks. Furthermore, radiation led to an increase in left ventricular end‐diastolic internal diameter (LVIDd) and left ventricular end‐systolic internal diameter (LVIDs) (Figure 1A; Table 1). These structural defects were accompanied by enlarged cardiomyocytes (Figure S1A). The overall cardiac dysfunctions were more pronounced at a dose of 20 Gy than when compared to that of 10 Gy (Figures 1A and S1A).

**TABLE 1:** Heart functional parameters of mice 4 weeks after irradiation.

Parameters	Irradiation		
	0 Gy	10 Gy	20 Gy
HR	547.80 ± 26.47	564.00 ± 21.32	557.40 ± 24.07
EF (%)	70.07 ± 2.02	51.72 ± 1.50***	44.67 ± 1.98^#^
FS (%)	34.32 ± 1.59	22.42 ± 0.82***	18.70 ± 1.00^#^
LVIDd (mm)	3.83 ± 0.08	3.98 ± 0.09	4.10 ± 0.05*
LVIDs (mm)	2.72 ± 0.08	3.08 ± 0.08**	3.25 ± 0.10**
LVPWd (mm)	0.83 ± 0.02	0.65 ± 0.02	0.67 ± 0.03
LVPWs (mm)	1.45 ± 0.06	0.98 ± 0.03	1.08 ± 0.05

*Note:* Heart function parameters were assessed using echocardiography (*n* = 6 for each group). Heart rate (HR), ejection fraction (EF), fraction of shortening (FS), left ventricular end‐diastolic internal diameter (LVIDd), left ventricular end‐systolic internal diameter (LVIDs), left ventricular end‐systolic posterior wall thickness (LVPWs), and left ventricular end‐systolic posterior wall thickness (LVPWs), were assessed. Data are expressed as mean ± SEM. All results were analyzed using one‐way ANOVA with Tukey's post hoc test and confirmed by three independent experiments.

*** *p* < 0.001.

** *p* < 0.01.

* *p* < 0.05 versus Ctrl.

^#^ *p* < 0.05 versus 10 Gy.

Masson, Sirius red staining of cardiac tissue sections was performed to analyze the degree of cardiac fibrosis, which showed that its degree increased with increasing irradiation dose, and 20 Gy was more fibrotic compared to 10 Gy (Figure 1B,C). The protein expression levels of α‐SMA, collagen I, and TGF‐β1 gradually increased in the heart tissue and CFs with the increase of irradiation dose (Figure 1D,E). Further immunofluorescence staining of heart sections showed that perivascular fibrosis worsened with increasing radiation dose (Figure S2A).

Additionally, immunofluorescence staining of CFs showed that the fluorescence intensity of α‐SMA in cardiac fibroblasts gradually increased with a higher radiation dose, indicating an increase in the degree of fibrosis and transdifferentiation of CFs into myofibroblasts (Figure S2B).

### 3.2 Radiation Induces METTL3‐Mediated m6A Modification

**FIGURE 2:**
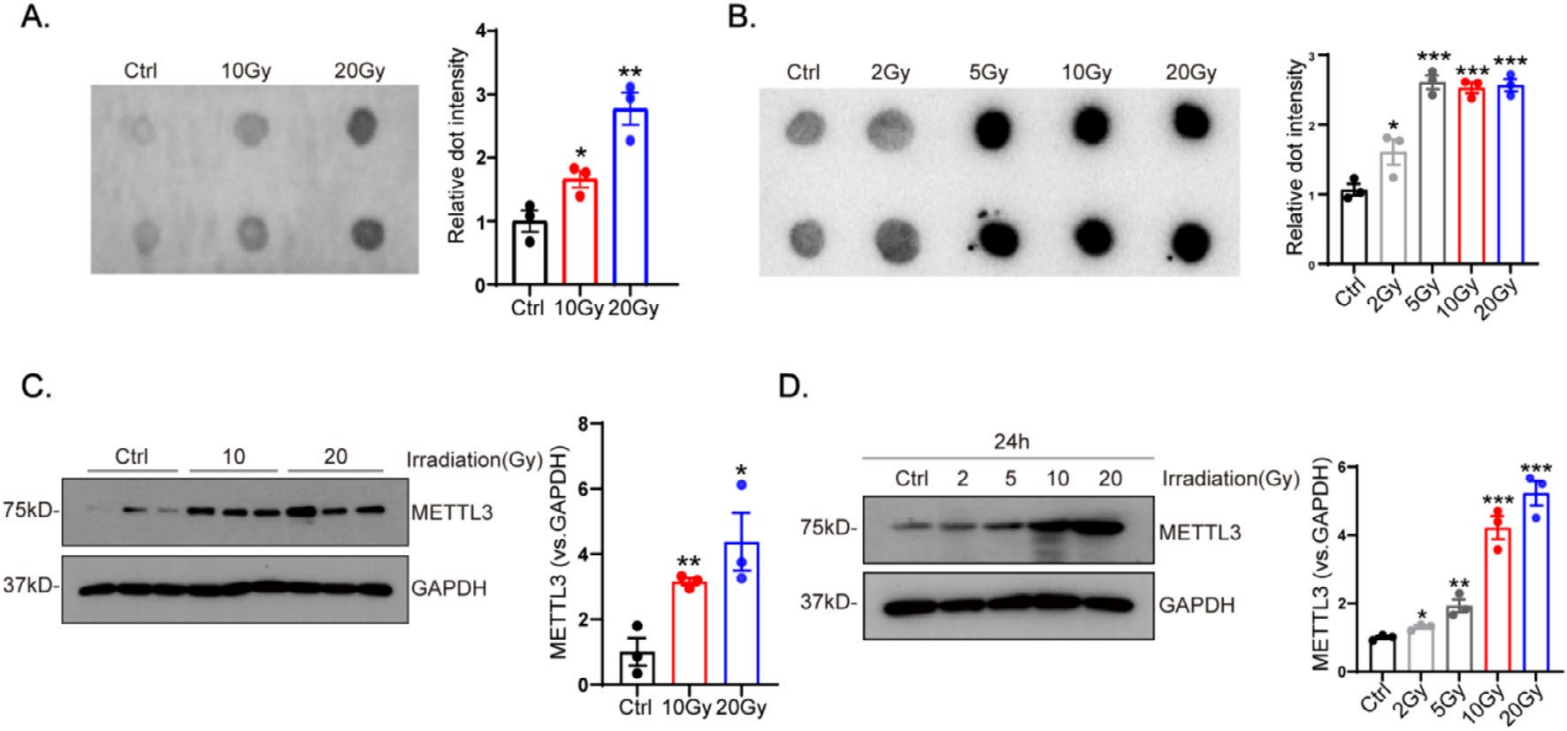
Radiation induces METTL3‐mediated m6A modification of mRNA. Adult mice and cardiac fibroblasts were subjected to irradiation at doses of 0 (Ctrl), 2, 5, 10, and 20 Gy, with exposure for 4 weeks and 24 h. m6A RNA modification in heart tissues (A) and cardiac fibroblasts (B) were measured using dot blot assay (*n* = 3). METTL3 expression in heart tissues (C) and cardiac fibroblast (D) were also quantified using western blot (*n* = 3). Data are shown as mean ± SEM. Results were analyzed using one‐way ANOVA with Tukey's *post hoc* test and confirmed by three independent experiments. ***p < 0.001, **p < 0.01, *p < 0.05 versus Ctrl.

A dot blot assay of cardiac tissues revealed that CFs of the irradiated group exhibited a significantly higher level of m6A modification in total RNA than that of the control group (Figure 2A,B). Similarly, a dose‐dependent increase in METTL3 levels was also demonstrated by western blot in cardiac tissues (Figure 2C). Consistent with the findings in heart tissue, cardiac fibroblasts exposed to X‐rays at varying doses (0, 2, 5, 10, and 20 Gy) showed a marked, dose‐dependent increase in METTL3 expression (Figure 2D).

### 3.3 METTL3 Is Key to Impaired Heart Function and Myocardial Fibrosis During Irradiation

**FIGURE 3:**
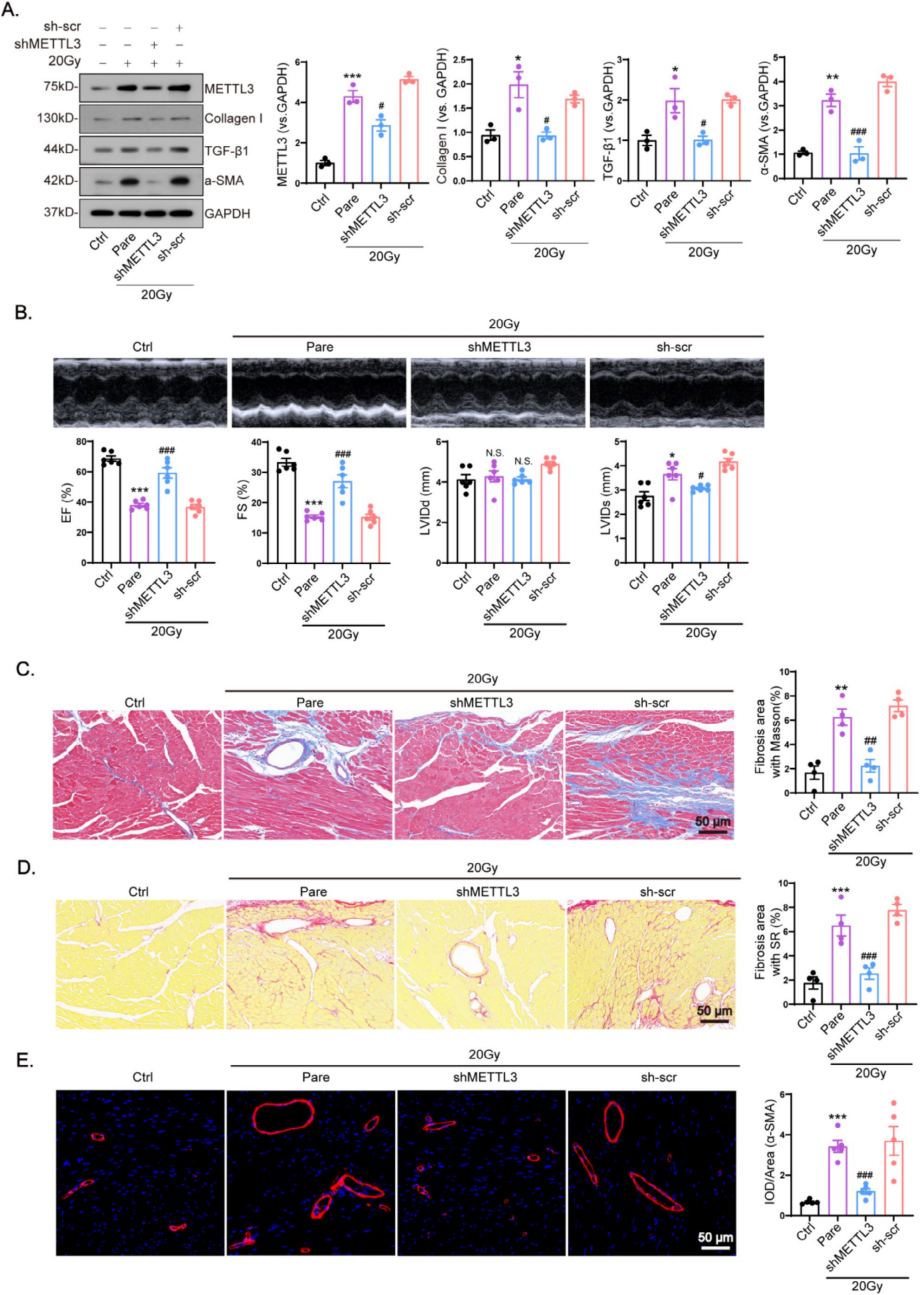
METTL3 is key to impaired heart function and myocardial fibrosis during irradiation. Adult mice were subjected to irradiation at doses of 0 Gy (Ctrl), 20 Gy (Pare), 20 Gy with cardiac co‐transfection of shMETTL3 or sh‐vector (sh‐scr) for 4 weeks. (A) Expression of METTL3 and fibrotic markers including α‐SMA, collagen I, and TGF‐β1 were quantified using western blot in mice heart (*n* = 3). (B) Heart function parameters including left ventricular ejection fraction, left ventricular fractional shortening, left ventricular internal diameter, and left ventricular posterior wall thickness, were assessed using echocardiography (*n* = 6). (C, D) Tissue fibrosis was evaluated using Masson and Sirius red staining (*n* = 4). (E) Myocardial fibrosis was evaluated using fluorescence intensity and representative images of α‐SMA fluorescence intensity enhancement in irradiated hearts after METTL3 inhibition(*n* = 5). Data are shown as mean ± SEM. Results were analyzed using one‐way ANOVA with Tukey's post hoc test. ***p < 0.001, **p < 0.01, *p < 0.05 versus Ctrl; ^###^ *p* < 0.001, ^##^ *p* < 0.01, ^#^ *p* < 0.05 versus Pare.

To investigate the effects of METTL3 inhibition on myocardial fibrosis and cardiac function following X‐ray irradiation, lentiviral vectors encoding shMETTL3 were injected into mouse hearts 2 weeks before irradiation. Western blot analysis showed that irradiated hearts exhibited more than a fivefold decrease in METTL3 expression compared to control hearts, while blocking METTL3 significantly decreased the levels of TGF‐β1, Collagen I, and α‐SMA in hearts exposed to radiation (Figure 3A). Echocardiographic analysis was performed after 4 weeks, and the results showed that LVEF and FS decreased significantly, and LVIDs increased after irradiation. However, the injection of shMETTL3 preserved normal cardiac function in radiation‐exposed mice, with no significant change in the LVEF, FS, and LVID values when compared with the control group (Figure 3B; Table 2).

**TABLE 2:** Heart function of irradiated adult mice with cardiac co‐transduction of shMETTL3 or scrambled shRNA (sh‐scr) for 4 weeks.

Parameters	Treatment			
	Ctrl	Pare	shMETTL3	sh‐scr
HR	542.00 ± 26.81	582.00 ± 24.99	567.00 ± 21.46	565.6 ± 20.97
EF (%)	68.67 ± 1.75	37.97 ± 1.14***	59.20 ± 3.37^###^	36.77 ± 1.64
FS (%)	33.32 ± 1.29	15.40 ± 0.55***	27.05 ± 2.09^###^	14.92 ± 0.76
LVIDd (mm)	4.13 ± 0.23	4.28 ± 0.28	4.15 ± 0.11	4.90 ± 0.11
LVIDs (mm)	2.75 ± 0.18	3.65 ± 0.23*	3.07 ± 0.05^#^	4.17 ± 0.13
LVPWd (mm)	0.75 ± 0.02	0.70 ± 0.04	0.67 ± 0.03	0.68 ± 0.05
LVPWs (mm)	1.18 ± 0.05	0.97 ± 0.08	1.00 ± 0.07	0.97 ± 0.10

*Note:* Adult mice were subjected to irradiation at doses of 0 Gy (Ctrl), 20 Gy (Pare) and 20 Gy with cardiac co‐transduction of shMETTL3 or scramble shRNA (sh‐scr) for 4 weeks (*n* = 6 for each group). Data are expressed as mean ± standard deviation. All results were analyzed using one‐way ANOVA with Tukey's *post hoc* test and confirmed by three independent experiments.

*** *p* < 0.001.

* *p* < 0.05 versus Ctrl.

^###^ *p* < 0.001.

^#^ *p* < 0.05 versus Pare.

Interstitial fibrosis was assessed 4 weeks post‐irradiation. METTL3 inhibition reduced interstitial and perivascular collagen deposition in irradiated myocardial tissues (Figure 3C,D). Additionally, shMETTL3 treatment reduces the fluorescence intensity of the irradiated cardiac myofibroblast marker α‐SMA (Figure 3E). In addition, cardiomyocyte area increased after 20 Gy irradiation, but significantly decreased after shMETTL3 virus injection (Figure S3A). STM2457, an inhibitor of METTL3, was used to recognize the role of METTL3 in the post‐irradiated heart. Following the study protocol shown in (Figure S4A), 8‐week‐old mice were irradiated with daily tail vein injections of STM2457 (25 mg/kg). Western blot results showed that injection of STM2457 successfully down‐regulated METTL3 levels and reduced the levels of TGF‐β1, collagen I, and α‐SMA in irradiated hearts (Figure S4B,C). Meanwhile, STM2457 significantly improved LVEF and FS values and reduced LVIDs (Figure S4D, Table 3) compared with the control group. Also, injection of STM2457 reduced irradiated myocardial tissue interstitium (Figure S4E,F) and perivascular collagen deposition (Figure S4G). These data clearly demonstrate that blockade of METTL3 is effective in preventing myocardial fibrosis and attenuating X‐ray irradiation‐induced cardiac remodeling.

**TABLE 3:** Heart function in irradiated adult mice after 4 weeks of treatment with STM2457.

Parameters	Treatment			
	Ctrl	Pare	STM2457	Veh
HR	542.00 ± 26.81	582.00 ± 24.99	567.00 ± 21.46	565.6 ± 20.97
EF (%)	64.14 ± 1.539	45.76 ± 2.24***	55.64 ± 1.32^##^	43.70 ± 0.93
FS (%)	31.10 ± 0.87	20.76 ± 0.70***	30.26 ± 1.13^###^	22.00 ± 0.58
LVIDd (mm)	3.640 ± 0.23	3.840 ± 0.10	3.720 ± 0.10	3.820 ± 0.11
LVIDs (mm)	2.760 ± 0.09	3.360 ± 0.07*	2.720 ± 0.07^#^	3.020 ± 0.11
LVPWd (mm)	0.71 ± 0.03	0.69 ± 0.04	0.66 ± 0.02	0.65 ± 0.05
LVPWs (mm)	1.02 ± 0.05	1.08 ± 0.08	0.98 ± 0.06	0.97 ± 0.10

*Note:* Adult mice were subjected to irradiation at doses of 0 Gy (Ctrl), 20 Gy (Pare) and 20 Gy with STM2457 or PBS (Veh) for 4 weeks (*n* = 5 for each group). Data are expressed as mean ± standard deviation. All results were analyzed using one‐way ANOVA with Tukey's *post hoc* test and confirmed by three independent experiments.

*** *p* < 0.001.

* *p* < 0.05 versus Ctrl.

^###^ *p* < 0.001.

^##^ *p* < 0.01.

^#^ *p* < 0.05 versus Pare.

### 3.4 METTL3 Promotes the Transdifferentiation of Cardiac Fibroblasts to Myofibroblasts During Irradiation

CFs were transduced with plasmids to reduce the expression of METTL3. Western blot results showed that the expression level of METTL3 in the knockdown group was significantly reduced compared with that in the control group (Figure S5A) and that the knockdown of METTL3 also reduced the expression of α‐SMA, collagen I, and TGF‐β1 in CFs (Figure S5A). In the EDU assay, the knockdown of METTL3 was found to inhibit CF proliferation (Figure S6A), and the migration of CFs was inhibited in the transwell assay at 24 h after transduction (Figure S6B).

The overexpression of METTL3 increased α‐SMA, collagen I, and TGF‐β1 expression in CFs (Figure S5B) and reduced CFs proliferation in EDU experiments (Figure S6C) and migration in permeabilized‐well experiments at 24 h post‐transduction (Figure S6D). Immunofluorescence analysis further confirmed that the expression of the myofibroblast marker α‐SMA was increased in the METTL3 overexpression group, consistent with the results of the western blot (Figure S6E).

**FIGURE 4:**
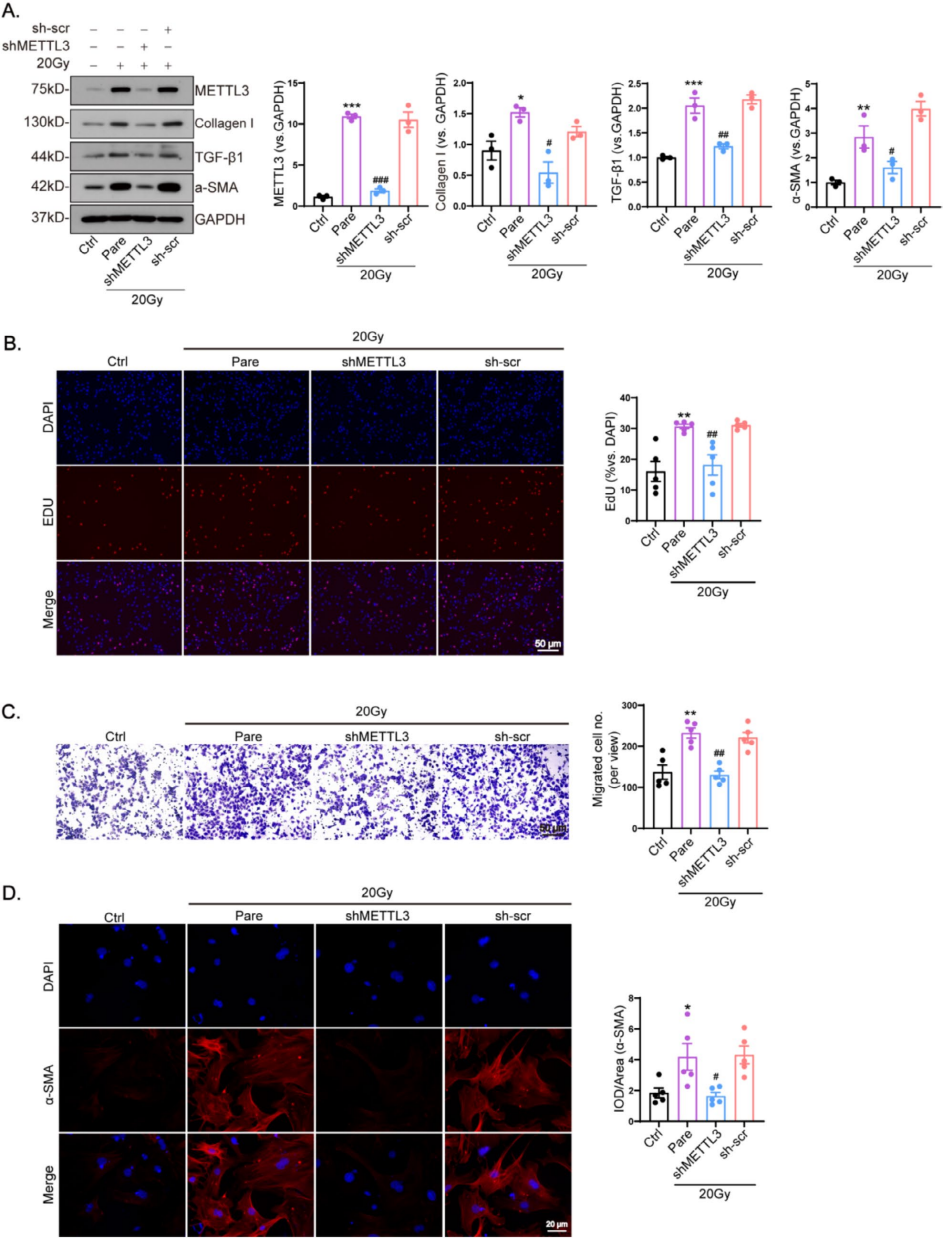
METTL3 modulates the transdifferentiation of cardiac fibroblasts into myofibroblasts during irradiation. Cardiac fibroblasts were subjected to irradiation at doses of 0 Gy (Ctrl), 20 Gy (Pare), 20 Gy with co‐transduction of shMETTL3 (shMETTL3) or scramble shRNA(sh‐scr) for 24 h. (A) Expression of METTL3 and myofibroblast markers including α‐SMA, collagen I, and TGF‐β1 were quantified using western blot (*n* = 3). Representative images and statistical analysis of shMETTL3 inhibition of radiation‐induced CF proliferation (B) and migration (C) transdifferentiation to myofibroblasts (D) were also evaluated using fluorescence intensity(*n* = 5). Data are shown as mean ± SEM. Results were analyzed using one‐way ANOVA with Tukey's *post hoc* test. ***p < 0.001, **p < 0.01, *p < 0.05 versus Ctrl; ^###^ *p* < 0.001, ^##^ *p* < 0.01, ^#^ *p* < 0.05 versus Pare.

CFs were transducted with shMETTL3 or the corresponding negative viruses. shMETTL3 effectively decreased METTL3 expression in irradiated CFs compared to the control group (Figure 4A). Furthermore, X‐ray irradiation suppressed the expression of α‐SMA, Collagen I, and TGF‐β1 (Figure 4A). Irradiation significantly enhanced the proliferation and migration of CFs, but transfection with shMETTL3 substantially attenuated these radiation‐induced effects (Figure 4B,C). Moreover, immunofluorescence analysis revealed that shMETTL3 treatment reduced the fluorescence intensity of CFs post‐irradiation (Figure 4D), indicating that METTL3 knockdown impeded myofibroblast differentiation in response to radiation.

The METTL3 inhibitor STM2457 also imitated these effects. First, we tested the optimal concentration of STM2457 in cardiac fibroblasts by treating CF with 0, 5, 10, 15, and 20 μM, respectively. The cell viability was maintained at around 100% when the concentration of STM 2457 was lower than 5 μM, which was drastically reduced to 78.86% and 56.58% by 10 and 15 μM, respectively (Figure S7A). In radiation‐treated CF, the relative total methylation level was significantly increased but significantly decreased after treatment with 5 μM STM 2457 (Figure S7B). Following STM2457 pretreatment, western blot analysis showed a significant reduction in METTL3 expression and a corresponding decrease in α‐SMA, Collagen I, and TGF‐β1 expression in irradiated CFs (Figure S7C,D). STM2457 pretreatment also inhibited CF proliferation (Figure S7E) and migration (Figure S7F). Immunofluorescence analysis further confirmed that the expression of the myofibroblast marker α‐SMA was significantly decreased after STM2457 pretreatment (Figure S7G).

### 3.5 Cardiac METTL3 Regulates the Akt/mTOR Signaling Pathway in Irradiation

Thirty‐six mouse heart samples were grouped according to high or low expression of METTL3, and 43 up‐regulated differential genes were identified in the radiological dataset GSE218447. KEGG enrichment analysis revealed that the PI3K‐Akt pathway was significantly enriched (Figure S8A). Thus, we hypothesized that the PI3K‐Akt pathway plays a key role in the regulation of radiation‐induced heart disease by METTL3.

**FIGURE 5:**
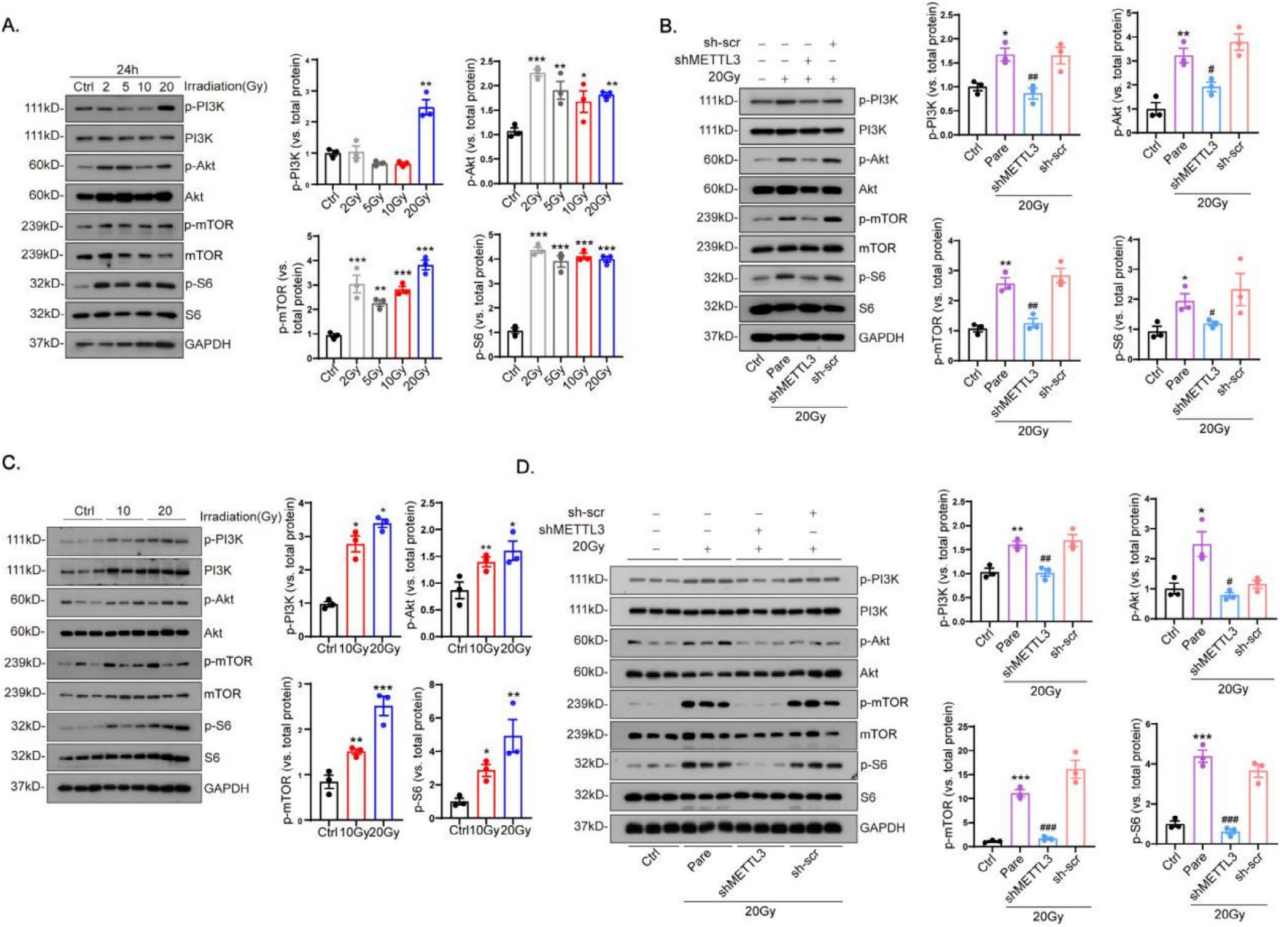
METTL3 initiates the Akt–mTOR cascades during irradiation. Adult mice and cardiac fibroblasts were subjected to irradiation at doses of 0 Gy (Ctrl), 2 Gy, 5 Gy, 20 Gy (Pare), 20 Gy with cardiac co‐transduction of shMETTL3 or scramble shRNA (sh‐scr) and exposure for 4 weeks and 24 h, respectively. Phosphorylation of signaling molecules including PI3K, Akt, S6 and mTOR in heart tissues (A, B) and cardiac fibroblasts (C, D) was quantified using western blot (*n* = 3). Data are shown as mean ± SEM. Results were analyzed using one‐way ANOVA with Tukey's post hoc test and confirmed by three independent experiments. ***p < 0.001, **p < 0.01, *p < 0.05 versus Ctrl; ^###^ *p* < 0.001, ^##^ *p* < 0.01, ^#^ *p* < 0.05 versus Pare.

Western blot analysis demonstrated that the phosphorylation levels of mTOR and Akt increase in a dose‐dependent manner with increased radiation in CFs (Figure 5A). Alternatively, the phosphorylation levels of mTOR and Akt decreased in irradiated CFs following transduction with shMETTL3 (Figure 5B). Phosphorylation levels of mTOR and Akt also decreased after METTL3 knockdown (Figure S9A) and increased after METTL3 overexpression (Figure S9B).

Mice were transfected with shMETTL3 or sh‐scr viruses, and CFs were extracted from mouse hearts after different doses of irradiation. Subsequent western blot analysis confirmed enhanced phosphorylation levels of Akt and downstream signaling in cardiac CFs after this irradiation (Figure 5C). However, in irradiated CFs transfected with shMETTL3, phosphorylation levels of Akt and downstream signaling were significantly reduced (Figure 5D).

### 3.6 METTL3 Catalyzes Akt mRNA m6A Modification and Enhances Its Stability Through IGF2BP1

**FIGURE 6:**
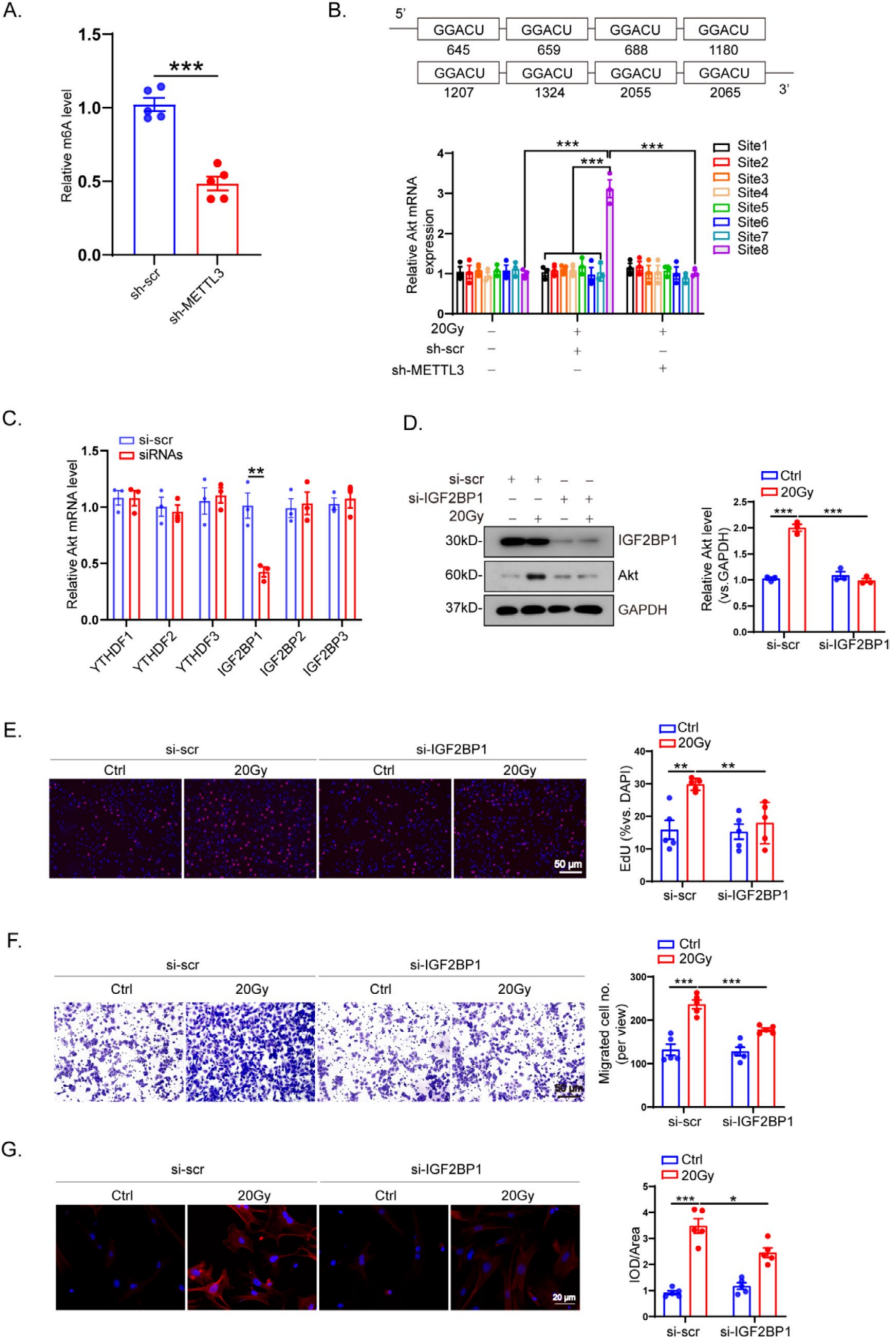
Akt serves as a target for METTL3‐mediated m6A modification via an IGF2BP1‐dependent manner. (A) Representative m6A dot blot and statistical analysis of m6A with or without METTL3 knockdown (*n* = 5). (B) MeRIP‐qPCR analysis showing the location of given m6A methylation sites in Akt (*n* = 3). (C) Relative mRNA levels of Akt in CFs knockdown with YTH and IGF2BP reader proteins (*n* = 3). (D) Relative protein level of p‐Akt in the absence or presence of IGF2BP1 knockdown and quantification (*n* = 3). Representative images and quantitation of cardiac fibroblasts with or without IGF2BP2 knockdown that proliferate (E), migrate (F), and differentiate into myofibroblasts (G) after receiving 20 Gy irradiation (*n* = 5). ***p < 0.001, **p < 0.01, *p < 0.05.

After knocking down METTL3, the phosphorylation levels of Akt and its downstream signaling were significantly reduced. Next, we investigated how METTL3 regulates Akt expression in radiation‐treated cardiac fibroblasts. We wondered whether the changes in Akt phosphorylation levels were attributable to m6A modification. First, we found that knocking down METTL3 significantly reduced m6A modification in cardiac fibroblasts (Figure 6A).

Next, potential m6A binding sites for Akt mRNA were predicted using the SRAMP database (http://www.cuilab.cn/sramp). We validated 25 potential m6A sites located on the Akt mRNA sequence (Figure S10A and Table S5). Of these sites, eight were predicted with very high confidence (Figure 6B). meRIP‐qPCR assays showed that radiation‐induced substantial hypermethylation of Akt mRNA at site 2065 which decreased significantly after METTL3 knockdown (Figure 6B). Similarly, treatment with the METTL3 inhibitor STM2457 also significantly decreased the m6A level of Akt (Figure S10B). These findings suggest that METTL3‐mediated modification of Akt m6A stabilizes its mRNA in radiation‐treated cardiac fibroblasts.

It is well known that m6A modifications have various functional effects on mRNAs by binding to m6A reader proteins [15]. To identify the m6A reader proteins responsible for the recognition of METTL3‐mediated m6A on Akt mRNA, key reader genes in cardiac fibroblasts were knocked down prior to radiation treatment, including YTHDF1, YTHDF2, YTHDF3, IGF2BP1, IGF2BP2, and IGF2BP3 (Figure S11A, Table S4). Knockdown of IGF2BP1, but not other readers, resulted in significant downregulation of Akt mRNA (Figure 6C), along with reduced phosphorylated Akt protein levels (Figure 6D). Furthermore, the knockdown of IGF2BP1 inhibited CF proliferation (Figure 6E), migration (Figure 6F), and fibroblast‐to‐myofibroblast transformation (Figure 6G), as well as cell viability (Figure S11B). These results suggest that METTL3‐mediated m6A modification promotes Akt mRNA stabilization through IGF2BP1 under radiation.

## 4 Discussion

**FIGURE 7:**
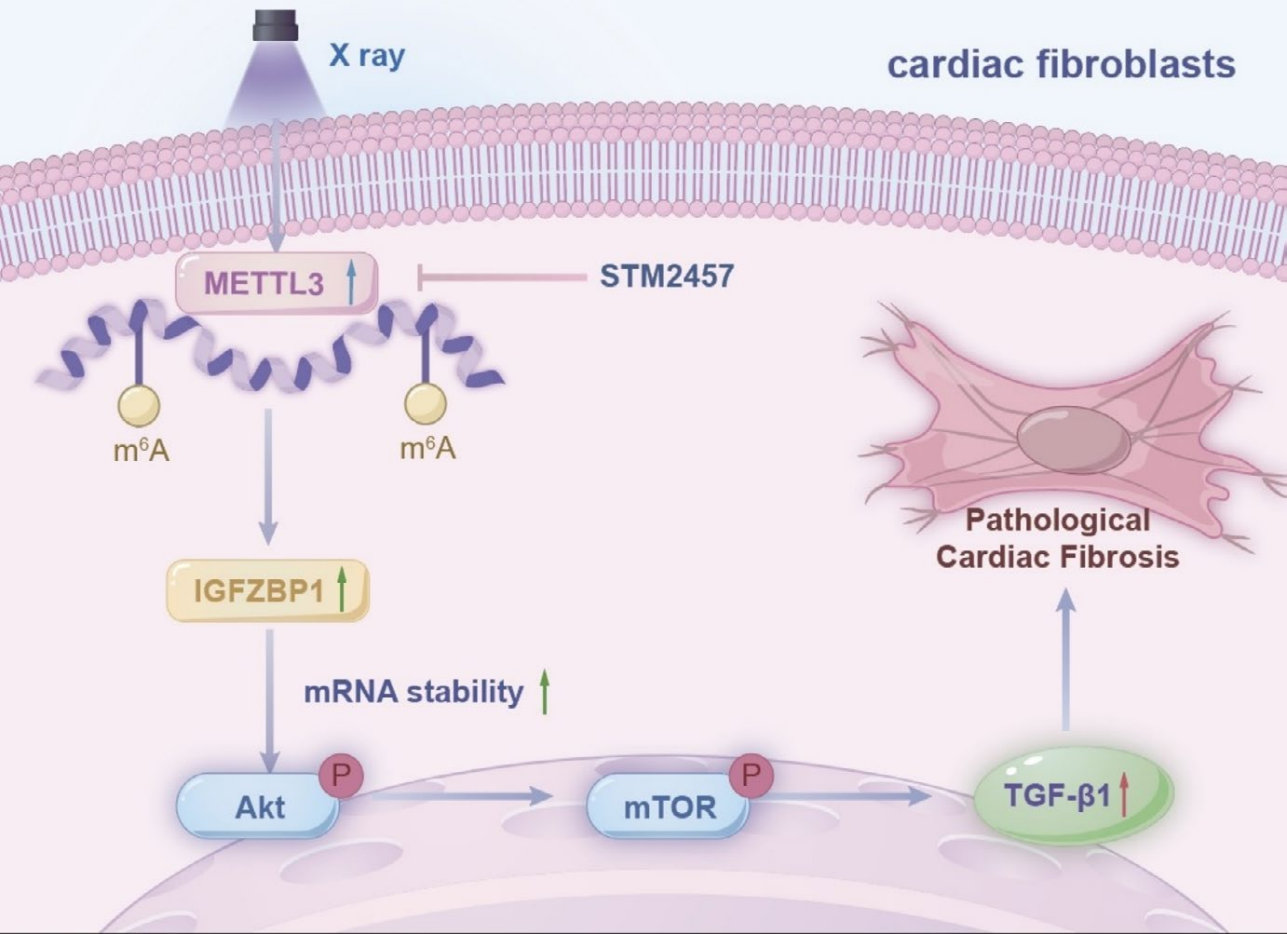
Effect of m6A RNA methylation‐regulated Akt/mTOR signaling pathway on radiation‐treated cardiac fibroblasts.

Our study establishes a previously unrecognized role of METTL3‐mediated m6A modification in radiation‐induced cardiotoxicity, demonstrating dose‐dependent upregulation of this epitranscriptomic regulator in both irradiated myocardium and activated cardiac fibroblasts. The central finding that pharmacological or genetic METTL3 inhibition significantly attenuates pro‐fibrotic signaling through Akt/mTOR axis modulation provides mechanistic insight into radiation‐associated fibrogenesis (Figure 7).

Current understanding posits that ionizing radiation induces biphasic cardiac remodeling, characterized by acute inflammatory responses followed by chronic fibrotic progression [13]. The dose–response relationship observed in our model (20 Gy‐induced collagen deposition more than 2.7‐fold) corroborates radiation cardiology studies showing structural compromise at clinically relevant doses (> 10 Gy) [16, 17]. Researchers found that increasing the radiation dose to 20 Gy led to cardiac fibrotic damage and structural damage [13]. The current study confirms that 20 Gy radiation leads to cardiomyocyte hypertrophy and myocardial fibrosis in mice, in parallel with similar results in CFs. Cardiac fibroblasts, which make up to 10%–25% of the total number of cells in the myocardium, have an essential role in maintaining structural and mechanical cardiac integrity [18]. In response to injury, CFs are activated and acquire a pro‐fibrotic phenotype commonly referred to as myofibroblasts, which is characterized by proliferative activity, excessive ECM production, and contractile function due to the expression of α‐SMA [8, 19, 20]. The current study corroborated these findings by demonstrating a dose‐dependent upregulation of the myofibroblast marker α‐SMA in CFs and mouse hearts post‐irradiation.

The paradoxical cardioprotective effects of low‐dose radiation (< 5 Gy) on ischemic remodeling stand in contrast to our demonstration of dose‐aggravated fibrosis at therapeutic radiation levels [21, 22, 23, 24]. However, this dichotomy suggests a biphasic dose–response relationship, where pro‐survival signaling dominates at subtoxic doses, while sustained epitranscriptomic stress drives maladaptive remodeling at higher exposures [25, 26]. Resolving this therapeutic window requires temporal single‐cell analyses to disentangle cell‐type‐specific radiation responses.

Emerging evidence positions m6A modification as a dynamic regulator of cardiac pathophysiology [27, 28]. While Mathiyalagan et al. [29] demonstrated the critical role of the FTO‐dependent cardiac m6A methylome in modulating heart failure and cardiac contraction, our work uniquely identifies METTL3 as a master regulator of radiation‐associated fibrogenesis. The current study revealed that METTL3 expression was up‐regulated in irradiated hearts, thereby intensifying cardiac fibrosis. Similar findings in irradiated CFs suggest a close association between METTL3 and RIHD. Notably, our demonstration that STM2457‐mediated METTL3 inhibition preserves ventricular mechanics highlights the translational potential for cardioprotective adjuvants in radiation oncology.

Studies have shown that Akt family members are expressed in the myocardium. Ma et al. [30] discovered that the cardioprotective effects of the pipeline were negated by the overexpression of constitutively active Akt. Inhibition of Akt significantly attenuated pressure‐overload‐induced cardiac fibrosis [31]. Previous studies have shown that mTOR is a direct substrate for AKT kinase. mTOR is also a serine/threonine kinase consisting of two complexes, mTORC1 and mTORC2 [32, 33]. Finckenberg et al. [34] found that when the mTOR pathway was blocked with everolimus in double‐transgenic rats, expression levels of the pro‐fibrotic factors TGF‐β1, Col I, and Col III were significantly down‐regulated. Mitra et al. [35] found that OSI‐027, a dual mTOR inhibitor, inhibited TGF‐β‐induced expression of α‐SMA, Col I, and Col III. Thus, AKT/mTOR may be involved in the regulation of cardiac fibrosis. Consistent with the above studies, it was found that the fibrosis level of CFs increased after irradiation and the phosphorylation levels of Akt, mTOR, and downstream S6 in CFs and cardiac tissues were up‐regulated according to the irradiation dose, while for knockdown of METTL3 both in vivo and in vitro the levels of phosphorylation of mTOR and downstream S6 went down. This suggests that the upregulation of METTL3 after irradiation exacerbates cardiac fibrosis, potentially through the AKT–mTOR pathway.

This study has several limitations. First, it should be noted that the expression of METTL3 is subject to dynamic regulation in response to varying levels of irradiation. However, the specific mechanisms that govern this regulation are still not well understood. Second, the induction of METTL3 in disease models is achieved by use of lentiviral vectors. However, the use of these vectors in clinical settings is limited due to the possibility of unintended effects on non‐target areas, constraints in delivery methods, and concerns over safety. Third, it has been discovered that specific molecular targets are influenced by METTL3 and play a role in regulating PI3K‐Akt phosphorylation. It is also important to thoroughly investigate additional molecules downstream that may possibly be involved in this process. Fourth, this experiment is reliant on healthy animal models, which limits its applicability and relevance and should be examined for broader effects in other disease states.

In conclusion, this investigation deciphers the mechanistic lexicon of METTL3 in radiation cardiotoxicity, positioning m6A methylation as a tunable epigenetic determinant of cardiac fibroblast activation. The therapeutic efficacy of STM2457 in preserving myocardial architecture provides proof of concept for epitranscriptome‐targeted interventions. Future efforts should optimize temporal–spatial METTL3 modulation while exploring combinatorial regimens with established radioprotectants.

## Author Contributions

Xiaosong Gu and Jing Zhu conceived the study and designed the study protocol. Shunsong Qiao and Jingjing Zhu constructed the animal model and collected samples. Chao Tang and Jing Zhu performed the echocardiographic analyses. Jingjing Zhu and Li Xiang conducted the literature review and statistical analysis. Shunsong Qiao and Li Xiang drafted the manuscript. Jingjing Zhu and Yu Feng reviewed the manuscript for intellectual content, making revisions as needed. All authors contributed to editorial changes in the manuscript and read and approved the final draft.

## Ethics Statement

All animal protocols adhered to the guidelines established by the Institutional Animal Care and Use Committee and the Ethics Committee of Soochow University.

## Consent

The authors have nothing to report.

## Conflicts of Interest

The authors declare no conflicts of interest.

## Supporting information


Figure S1.

Data S1.

Data S2.

Table S4.

## Data Availability

The datasets used and/or analyzed during the current study are available from the corresponding author on reasonable request.

## References

[1] P. M. Kostakou , N. T. Kouris , V. S. Kostopoulos , D. S. Damaskos , and C. D. Olympios , “Cardio‐Oncology: A New and Developing Sector of Research and Therapy in the Field of Cardiology,” Heart Failure Reviews 24, no. 1 (2019): 91–100. 30073443 10.1007/s10741-018-9731-y

[2] M. S. Lee , D. W. Liu , S. K. Hung , et al., “Emerging Challenges of Radiation‐Associated Cardiovascular Dysfunction (RACVD) in Modern Radiation Oncology: Clinical Practice, Bench Investigation, and Multidisciplinary Care,” Frontiers in Cardiovascular Medicine 7 (2020): 16. 32154267 10.3389/fcvm.2020.00016 PMC7047711

[3] B. Kura , B. Kalocayova , T. W. LeBaron , et al., “Regulation of MicroRNAs by Molecular Hydrogen Contributes to the Prevention of Radiation‐Induced Damage in the Rat Myocardium,” Molecular and Cellular Biochemistry 457, no. 1–2 (2019): 61–72. 30830529 10.1007/s11010-019-03512-z

[4] K. X. Wang , C. Ye , X. Yang , P. Ma , C. Yan , and L. Luo , “New Insights Into the Understanding of Mechanisms of Radiation‐Induced Heart Disease,” Current Treatment Options in Oncology 24, no. 1 (2023): 12–29. 36598620 10.1007/s11864-022-01041-4

[5] J. Yarnold and M. C. Brotons , “Pathogenetic Mechanisms in Radiation Fibrosis,” Radiotherapy and Oncology 97, no. 1 (2010): 149–161. 20888056 10.1016/j.radonc.2010.09.002

[6] M. Schlittler , P. P. Pramstaller , A. Rossini , and M. de Bortoli , “Myocardial Fibrosis in Hypertrophic Cardiomyopathy: A Perspective From Fibroblasts,” International Journal of Molecular Sciences 24, no. 19 (2023): 14845. 37834293 10.3390/ijms241914845 PMC10573356

[7] H. Guo , X. Zhao , H. Li , et al., “GDF15 Promotes Cardiac Fibrosis and Proliferation of Cardiac Fibroblasts via the MAPK/ERK1/2 Pathway After Irradiation in Rats,” Radiation Research 196, no. 2 (2021): 183–191. 34019665 10.1667/RADE-20-00206.1

[8] N. G. Frangogiannis , “Cardiac Fibrosis,” Cardiovascular Research 117, no. 6 (2021): 1450–1488. 33135058 10.1093/cvr/cvaa324 PMC8152700

[9] P. C. He and C. He , “M(6) A RNA Methylation: From Mechanisms to Therapeutic Potential,” EMBO Journal 40, no. 3 (2021): e105977. 33470439 10.15252/embj.2020105977 PMC7849164

[10] S. Wu , S. Zhang , X. Wu , and X. Zhou , “M(6)A RNA Methylation in Cardiovascular Diseases,” Molecular Therapy 28, no. 10 (2020): 2111–2119. 32910911 10.1016/j.ymthe.2020.08.010 PMC7544996

[11] J. N. Wang , F. Wang , J. Ke , et al., “Inhibition of METTL3 Attenuates Renal Injury and Inflammation by Alleviating TAB3 m6A Modifications via IGF2BP2‐Dependent Mechanisms,” Science Translational Medicine 14, no. 640 (2022): eabk2709. 35417191 10.1126/scitranslmed.abk2709

[12] S. Tapio , M. P. Little , J. C. Kaiser , et al., “Ionizing Radiation‐Induced Circulatory and Metabolic Diseases,” Environment International 146 (2021): 106235. 33157375 10.1016/j.envint.2020.106235 PMC10686049

[13] Y. Wu , L. Liu , S. Lv , et al., “Pyrrolidine Dithiocarbamate Might Mitigate Radiation‐Induced Heart Damage at an Early Stage in Rats,” Frontiers in Pharmacology 13 (2022): 832045. 35392554 10.3389/fphar.2022.832045 PMC8981468

[14] P. Yi , H. Li , J. Su , et al., “Trastuzumab Aggravates Radiation Induced Cardiotoxicity in Mice,” American Journal of Cancer Research 12, no. 1 (2022): 381–395. 35141025 PMC8822280

[15] K. Boulias and E. L. Greer , “Biological Roles of Adenine Methylation in RNA,” Nature Reviews Genetics 24, no. 3 (2023): 143–160. 10.1038/s41576-022-00534-0 PMC9974562 36261710

[16] L. Kiscsatari , M. Sárközy , B. Kővári , et al., “High‐Dose Radiation Induced Heart Damage in a Rat Model,” In Vivo 30, no. 5 (2016): 623–631. 27566082

[17] R. J. DeBo , C. J. Lees , G. O. Dugan , et al., “Late Effects of Total‐Body Gamma Irradiation on Cardiac Structure and Function in Male Rhesus Macaques,” Radiation Research 186, no. 1 (2016): 55–64. 27333082 10.1667/RR14357.1 PMC5068576

[18] M. Litvinukova , C. Talavera‐López , H. Maatz , et al., “Cells of the Adult Human Heart,” Nature 588, no. 7838 (2020): 466–472. 32971526 10.1038/s41586-020-2797-4 PMC7681775

[19] K. M. Herum , J. Choppe , A. Kumar , A. J. Engler , and A. D. McCulloch , “Mechanical Regulation of Cardiac Fibroblast Profibrotic Phenotypes,” Molecular Biology of the Cell 28, no. 14 (2017): 1871–1882. 28468977 10.1091/mbc.E17-01-0014 PMC5541838

[20] J. E. Baik , H. J. Park , R. P. Kataru , et al., “TGF‐Beta1 Mediates Pathologic Changes of Secondary Lymphedema by Promoting Fibrosis and Inflammation,” Clinical and Translational Medicine 12, no. 6 (2022): e758. 35652284 10.1002/ctm2.758 PMC9160979

[21] G. SenthilKumar , J. S. Heisner , R. Schlaak , et al., “Targeted Cardiac Ionizing Radiation in Dahl Salt‐Sensitive Rats Can Improve Recovery From Ischemic Injury,” JACC: Basic to Translational Science 8, no. 8 (2023): 1025–1027. 37719423 10.1016/j.jacbts.2023.07.004 PMC10504396

[22] L. N. Pedersen , C. V. Ripoll , M. Ozcan , et al., “Cardiac Radiation Improves Ventricular Function in Mice and Humans With Cardiomyopathy,” Med 4, no. 12 (2023): 928–943.e5. 38029754 10.1016/j.medj.2023.10.006 PMC10994563

[23] D. M. Zhang , R. Navara , T. Yin , et al., “Cardiac Radiotherapy Induces Electrical Conduction Reprogramming in the Absence of Transmural Fibrosis,” Nature Communications 12, no. 1 (2021): 5558. 10.1038/s41467-021-25730-0 PMC8463558 34561429

[24] F. Rodel , B. Frey , U. Gaipl , et al., “Modulation of Inflammatory Immune Reactions by Low‐Dose Ionizing Radiation: Molecular Mechanisms and Clinical Application,” Current Medicinal Chemistry 19, no. 12 (2012): 1741–1750. 22414082 10.2174/092986712800099866

[25] E. Shin , D. Kim , Y. Y. Choi , H. S. Youn , K. M. Seong , and B. H. Youn , “LDR‐Adapted Liver‐Derived Cytokines Have Potential to Induce Atherosclerosis,” International Journal of Radiation Biology 99, no. 5 (2023): 791–806. 36383216 10.1080/09553002.2023.2145028

[26] R. Ramadan , M. Claessens , E. Cocquyt , et al., “X‐Irradiation Induces Acute and Early Term Inflammatory Responses in Atherosclerosis‐Prone ApoE−/− Mice and in Endothelial Cells,” Molecular Medicine Reports 23, no. 6 (2021): 399. 33786610 10.3892/mmr.2021.12038 PMC8025474

[27] V. Kmietczyk , E. Riechert , L. Kalinski , et al., “M(6)A‐mRNA Methylation Regulates Cardiac Gene Expression and Cellular Growth,” Life Science Alliance 2, no. 2 (2019): e201800233. 30967445 10.26508/lsa.201800233 PMC6458851

[28] H. L. Sun , A. C. Zhu , Y. Gao , et al., “Stabilization of ERK‐Phosphorylated METTL3 by USP5 Increases m(6)A Methylation,” Molecular Cell 80, no. 4 (2020): 633–647.e7. 33217317 10.1016/j.molcel.2020.10.026 PMC7720844

[29] T. Wang , L. Y. Zhou , X. M. Li , et al., “ABRO1 Arrests Cardiomyocyte Proliferation and Myocardial Repair by Suppressing PSPH,” Molecular Therapy 31, no. 3 (2023): 847–865. 36639869 10.1016/j.ymthe.2023.01.011 PMC10014284

[30] L. Meng , Y. Lu , X. Wang , et al., “NPRC Deletion Attenuates Cardiac Fibrosis in Diabetic Mice by Activating PKA/PKG and Inhibiting TGF‐Beta1/Smad Pathways,” Science Advances 9, no. 31 (2023): eadd4222. 37531438 10.1126/sciadv.add4222 PMC10396312

[31] K. Miyazawa and K. Miyazono , “Regulation of TGF‐Beta Family Signaling by Inhibitory Smads,” Cold Spring Harbor Perspectives in Biology 9, no. 3 (2017): a022095. 27920040 10.1101/cshperspect.a022095 PMC5334261

[32] J. Li , F. Ge , S. Wuken , et al., “Zerumbone, a Humulane Sesquiterpene From Syringa Pinnatifolia, Attenuates Cardiac Fibrosis by Inhibiting of the TGF‐Beta1/Smad Signaling Pathway After Myocardial Infarction in Mice,” Phytomedicine 100 (2022): 154078. 35405613 10.1016/j.phymed.2022.154078

[33] P. Abeyrathna and Y. Su , “The Critical Role of Akt in Cardiovascular Function,” Vascular Pharmacology 74 (2015): 38–48. 26025205 10.1016/j.vph.2015.05.008 PMC4659756

[34] Y. Zhang , S. Liu , J. L. Ma , et al., “Apocynum Venetum Leaf Extract Alleviated Doxorubicin‐Induced Cardiotoxicity Through the AKT/Bcl‐2 Signaling Pathway,” Phytomedicine 94 (2022): 153815. 34781232 10.1016/j.phymed.2021.153815

[35] W. Y. Wei , Z. G. Ma , S. C. Xu , N. Zhang , and Q. Z. Tang , “Pioglitazone Protected Against Cardiac Hypertrophy via Inhibiting AKT/GSK3beta and MAPK Signaling Pathways,” PPAR Research 2016 (2016): 9174190. 27110236 10.1155/2016/9174190 PMC4826695

