# Supplementary material for: METTL3 Modulates Radiation‐Induced Cardiac Fibrosis via the Akt/mTOR Pathway
Source: FASEB J. 2025 Jun 5;39(11):e70666. doi: 10.1096/fj.202403143RRRR (PMC12139579; doi:10.1096/fj.202403143RRRR)
Supplement: Supplementary file 1 — Figure S1. [file FSB2-39-e70666-s003.docx]

Supplementary Figure 1:


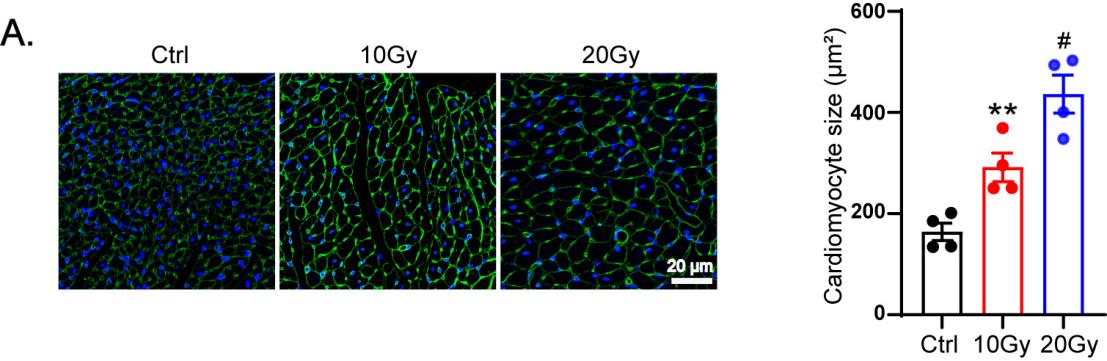


**Supplementary Figure 1:** Radiation increases the cardiomyocyte size. A. Adult mice were subjected to irradiation at doses of 0 Gy (Ctrl), 10 Gy and 20 Gy, with exposure for four weeks. The size of cardiomyocytes was measured using WGA staining (n=4 for each group). Data are expressed as mean ± SEM. Results were analyzed using one-way ANOVA with Tukey's *post hoc* test and confirmed by three independent experiments. ***p* < 0.01 vs. Ctrl; ^#^*p* < 0.05 vs. 10 Gy.

Supplementary Figure 2:


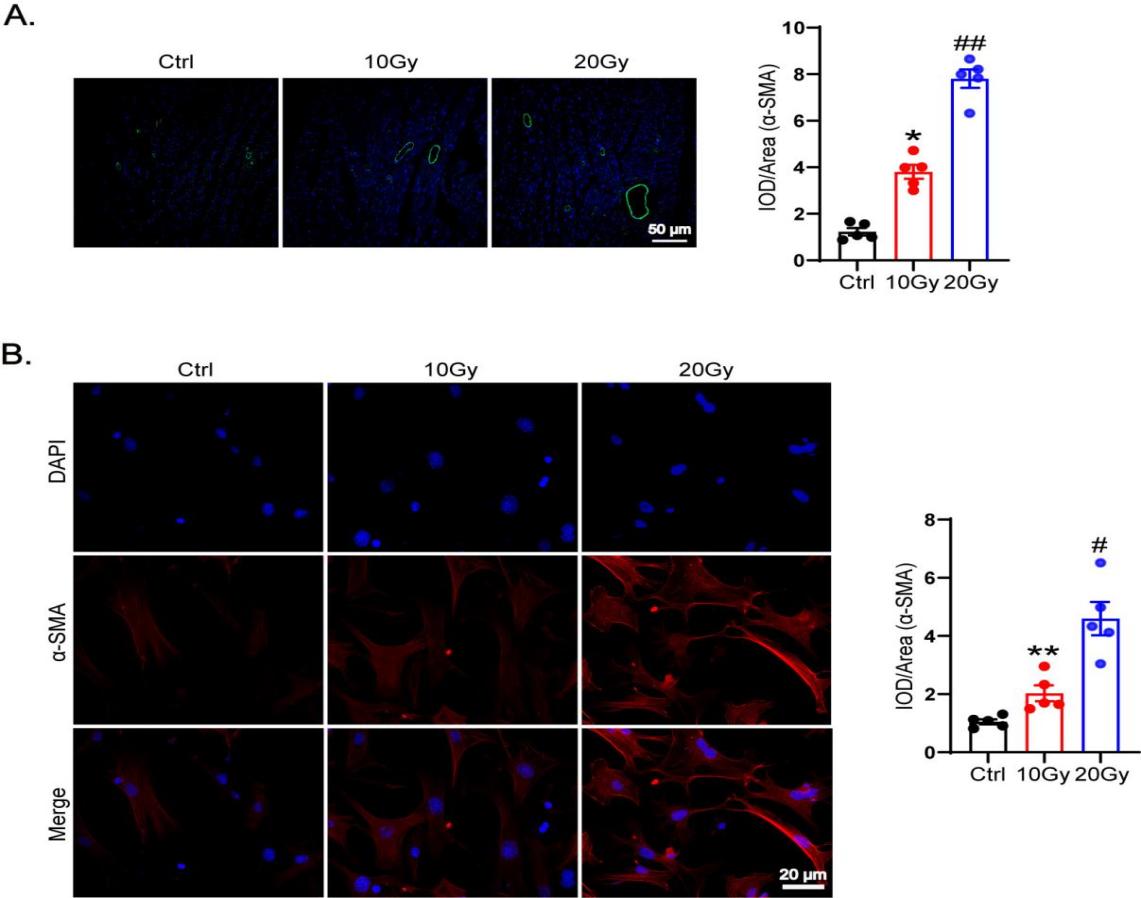


**Supplementary Figure 2:** Radiation is associated with increased transdifferentiation of cardiac fibroblasts into myofibroblasts. Adult mice and cardiac fibroblasts were subjected to irradiation at doses of 0 Gy (Ctrl), 10 Gy and 20 Gy, with exposure for four weeks and 24 hours, respectively. (A and B) The fibrotic marker α-SMA in heart tissues (A) and cardiac fibroblasts (B) were quantified using fluorescence intensity (n=5). Data are expressed as mean ± SEM. All results were analyzed using one-way ANOVA with Tukey's *post hoc* test and confirmed by three independent experiments. ***p* < 0.01, **p* < 0.05 vs. Ctrl; ^##^*p* < 0.01, ^#^*p* < 0.05 vs. 10 Gy.

Supplementary Figure 3:


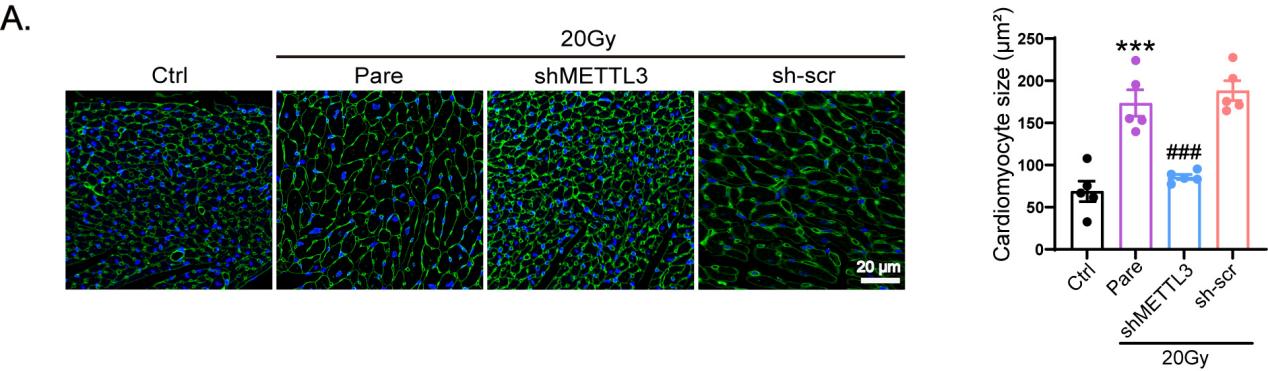


**Supplementary Figure 3:** METTL3 modulates the cardiomyocyte size during irradiation. Adult mice were subjected to irradiation at doses of 0Gy (control), 20Gy (Pare), 20Gy with cardiac co-transfection of shMETTL3(shMETTL3) or scramble shRNA(sh-scr) for four weeks（n=5 for each group）. The size of cardiomyocytes was quantified using WGA staining. Data are shown as mean±standard deviation. The results were analyzed using one-way ANOVA with Tukey’s post hoc test. ****p*<0.001 vs. Ctrl; ^###^*p*<0.001 vs. Pare.

Supplementary Figure 4:


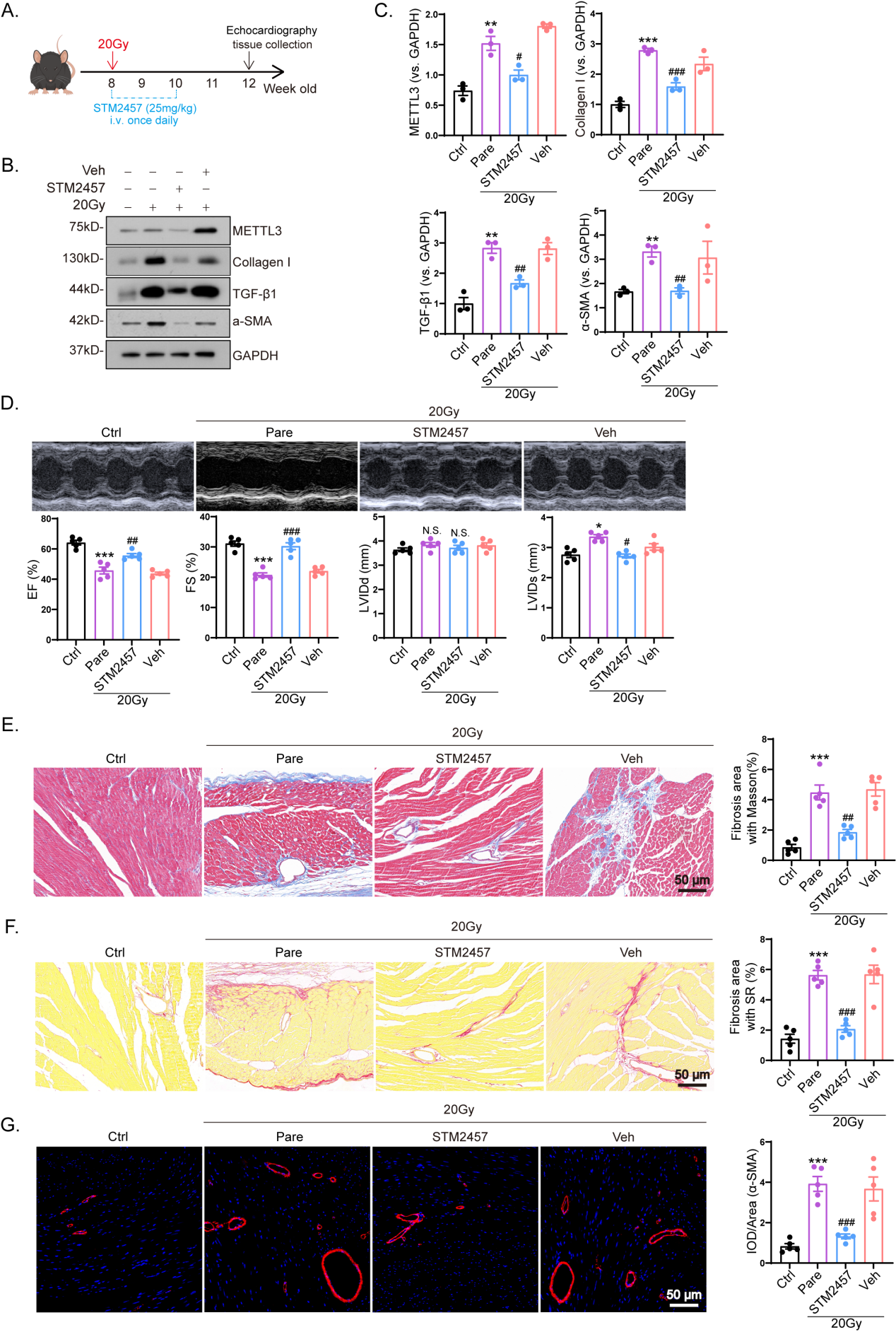


Supplementary Figure 4: Pharmacological inhibition of METTL3 protects against radiation-induced cardiac dysfunction and fibrosis. (A) Schematic schedule of irradiation and STM2457 treatment. Protein expression (B) and statistical analysis (C) of METTL3 and fibrosis markers including α-SMA, collagen I, and TGF-β1 in irradiated and STM2457-treated mouse hearts (n=3). (D) Representative M-mode echocardiograms and quantitative analysis. representative images of myocardial fibrosis assessed by Masson trichrome staining(E) ,Sirius red (F) and α-SMA fluorescence intensity (G) and quantitative analysis (n=5). Data are shown as mean±standard deviation. The results were analyzed using one-way ANOVA with Tukey’s post hoc test. ****p* < 0.001, ***p* < 0.01, **p* < 0.05 vs. Ctrl; ^##^*p* < 0.01, ^#^*p* < 0.05,^###^*p*<0.001 vs. Pare.

Supplementary Figure 5:


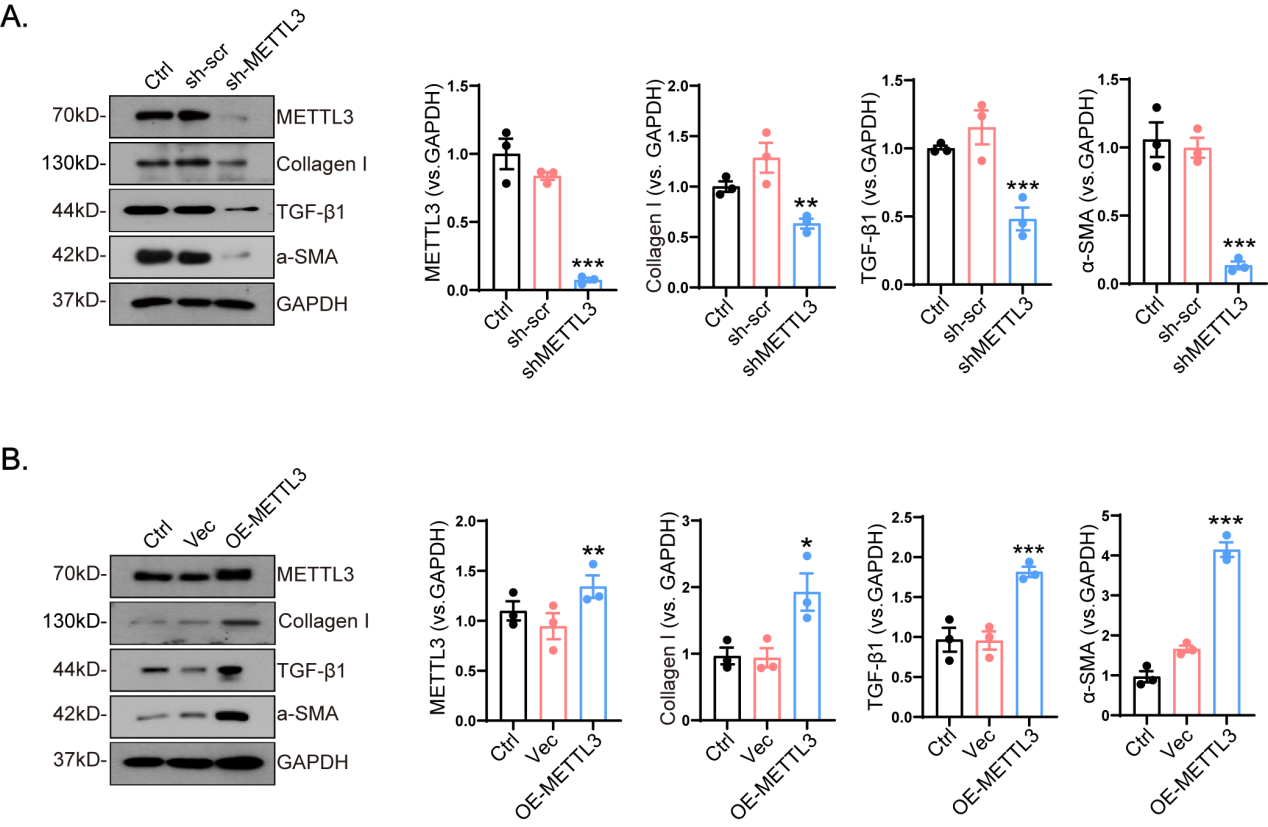


**Supplementary Figure 5:** METTL3 modulates the transdifferentiation of cardiac fibroblasts to myofibroblasts. (A) Cardiac fibroblasts were subjected to mock transduction (Ctrl), transduction of shMETTL3 or scramble shRNA (sh-scr) for 24 hours. Expression of METTL3 and myofibroblast markers including α-SMA, collagen I and TGF-β1 in were quantified using Western blot (n=3). (B) Cardiac fibroblasts were subjected to mock transduction (Ctrl), transduction of OE-METTL3 (shMETTL3) or sh-vector (Vec) for 24 hours, expression of METTL3 and the myofibroblast markers were likewise quantified using Western blot(n=3). Data are expressed as mean ± SEM. Results were analyzed using one-way ANOVA with Tukey's *post hoc* test. *** *p* < 0.001, ** *p* < 0.01, * *p* < 0.05 vs. Ctrl.

Supplementary Figure 6:


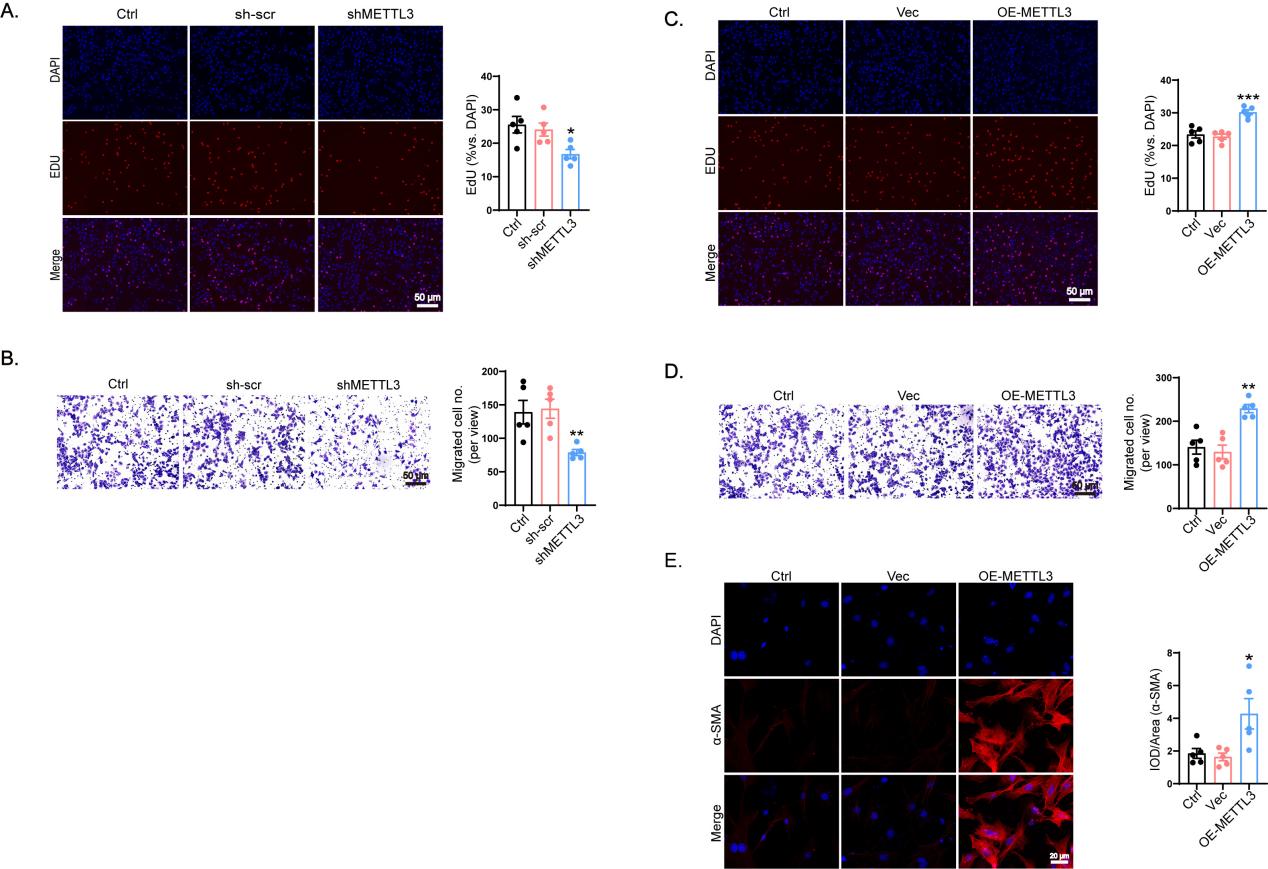


**Supplementary Figure 6:** METTL3 modulates cardiac fibroblast proliferation, migration and transdifferentiation into myofibroblasts. Cardiac fibroblasts were subjected to mock transduction (Ctrl), transduction of shMETTL3 (shMETTL3) or scramble shRNA (sh-scr) for 24 hours. (A and B) Cell proliferation and migration were evaluated using the EdU/DAPI ratio and the average number of migrating cells (n=5). Cardiac fibroblasts were subjected to mock transduction (Ctrl), transduction of OE-METTL3 (shMETTL3) or sh-vector (Vec) for 24 hours. (C,D and E) Cell proliferation, migration and transdifferentiation into myofibroblasts were similarly evaluated using fluorescence intensity(n=5). Data are expressed as mean ± SEM for groups of five mice. One-way ANOVA with Tukey's post hoc test was used for comparison between groups. ****p* < 0.001, ***p* < 0.01, **p* < 0.05 vs. Ctrl.

Supplementary Figure 7:


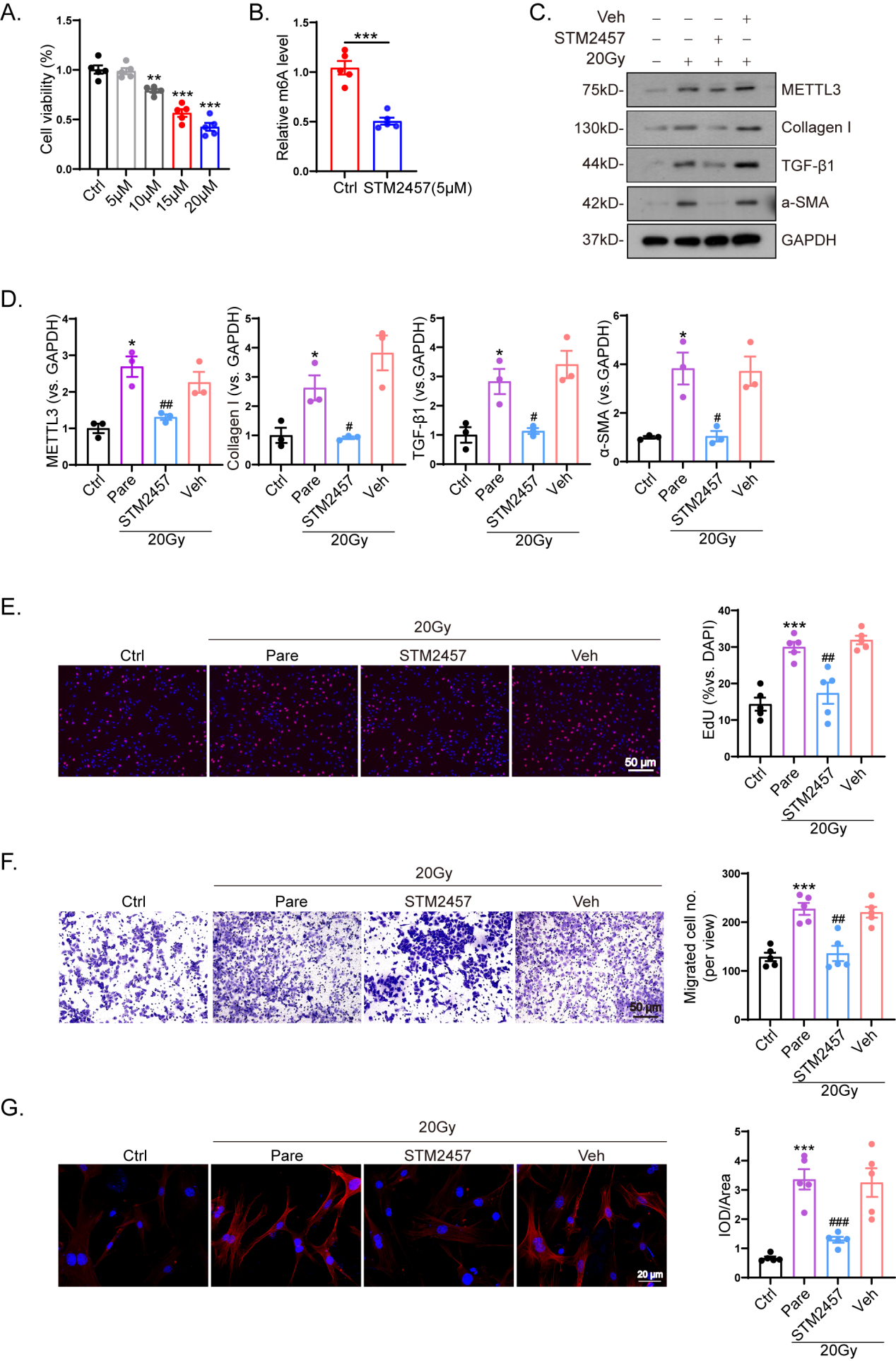


**Supplementary Figure 7:** STM 2457 attenuates irradiation (20 Gy)-stimulated transdifferentiation of cardiac primary fibroblasts (CF) to myofibroblasts. (A) CF were treated with 0, 5, 10, 15 and 20 μM STM 2457, and cell viability was detected by CCK-8 assay. (B) Relative total m6A methylation levels in STM 2457-treated CF were determined using the m6A RNA methylation quantification kit. Protein expression (C) and quantitative analysis (D) of METTL3 and myofibroblast markers including α-SMA, collagen I and TGF-β1 were detected using Western blot (n=3). Representative images and statistical analysis of STM 2457 inhibition of radiation-induced CF proliferation (E), migration (F) and transdifferentiation to myofibroblasts (G). Data are shown as mean±standard deviation. The results were analyzed using one-way ANOVA with Tukey’s post hoc test. ****p* < 0.001, ***p* < 0.01, **p* < 0.05 vs. Ctrl; ^##^*p* < 0.01, ^#^*p* < 0.05,^###^*p*<0.001 vs. Pare.

Supplementary Figure 8:


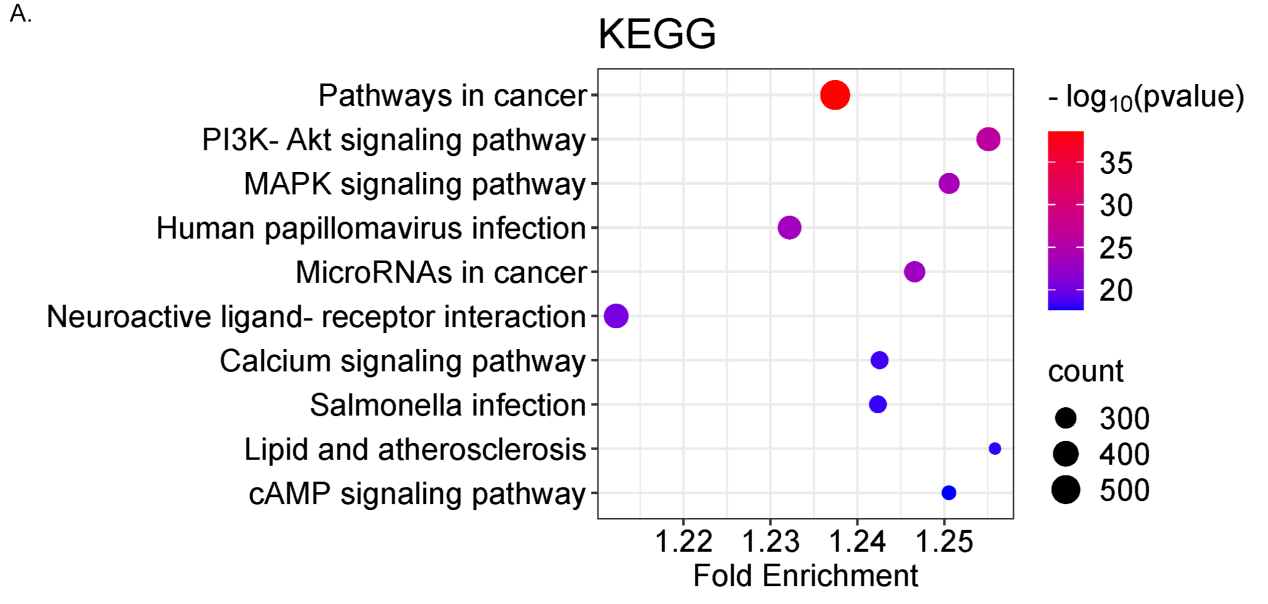


**Supplementary Figure 8:** Kyoto Encyclopedia of Genes and Genomes (KEGG) pathway enrichment analysis of METTL3-related genes during irradiation. A. Significantly enriched KEGG pathway analyses for 43 related genes that were differentially expressed in mouse heart tissues during irradiation.

Supplementary Figure 9:


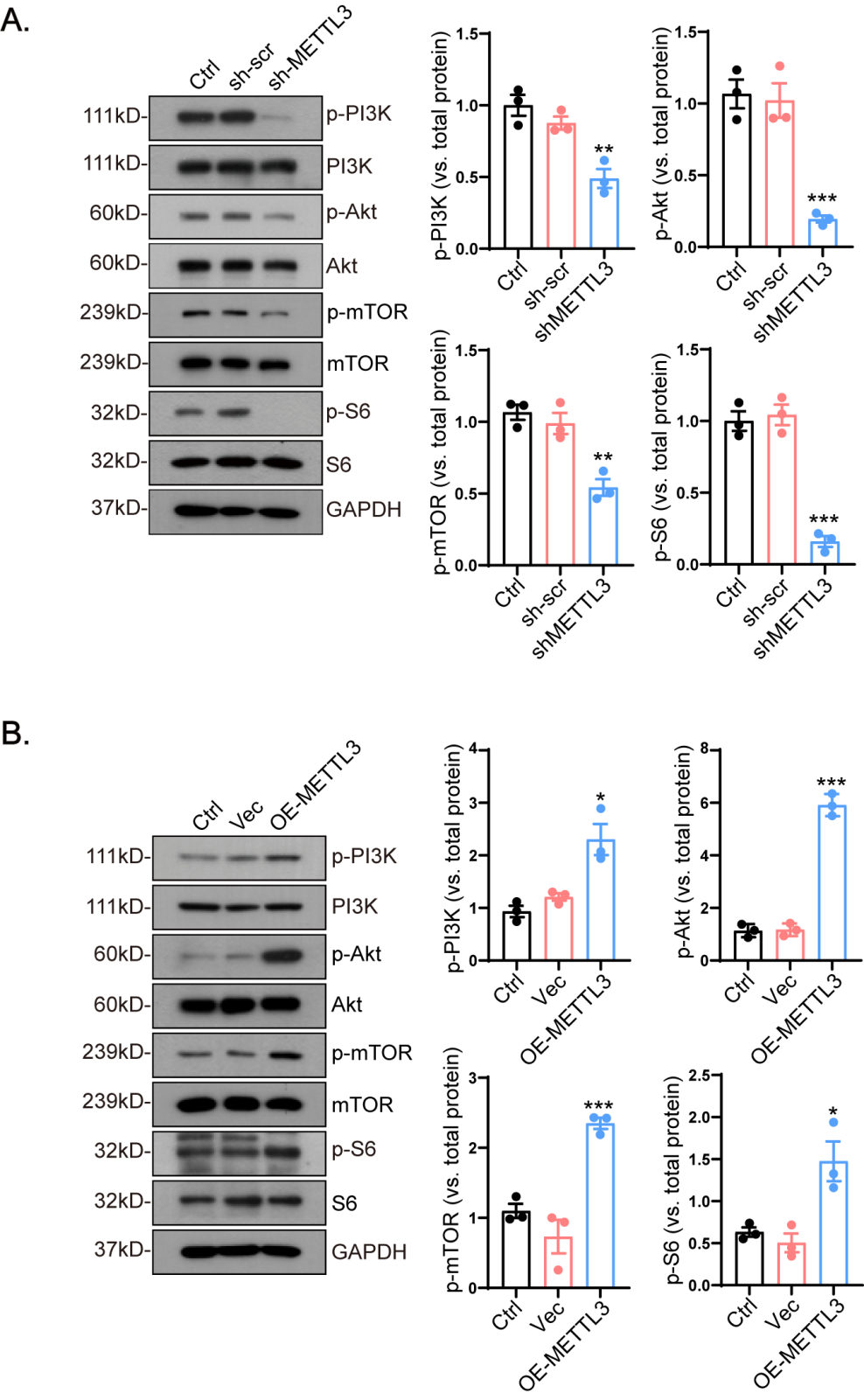


**Supplementary Figure 9:** METTL3 initiates the Akt-mTOR cascades in cardiac fibroblasts. (A) Cardiac fibroblasts were subjected to mock transduction (Ctrl), transduction of shMETTL3 or scramble shRNA (sh-scr) for 24 hours, phosphorylation of signaling molecules including PI3K, Akt, S6 and mTOR in cardiac fibroblasts was quantified using Western blot (n=3). (B) Cardiac fibroblasts were subjected to mock transduction (Ctrl), transduction of OE-METTL3 (shMETTL3) or sh-vector (Vec) for 24 hours, phosphorylation of the signaling molecules in the Akt-mTOR cascades were likewise quantified using Western blot (n=3). Data are expressed as mean ± SEM. Results were analyzed using one-way ANOVA with Tukey's *post hoc* test and confirmed by three independent experiments. ****p* < 0.001,***p* < 0.01,**p* < 0.05 vs. Ctrl.

Supplementary Figure 10:


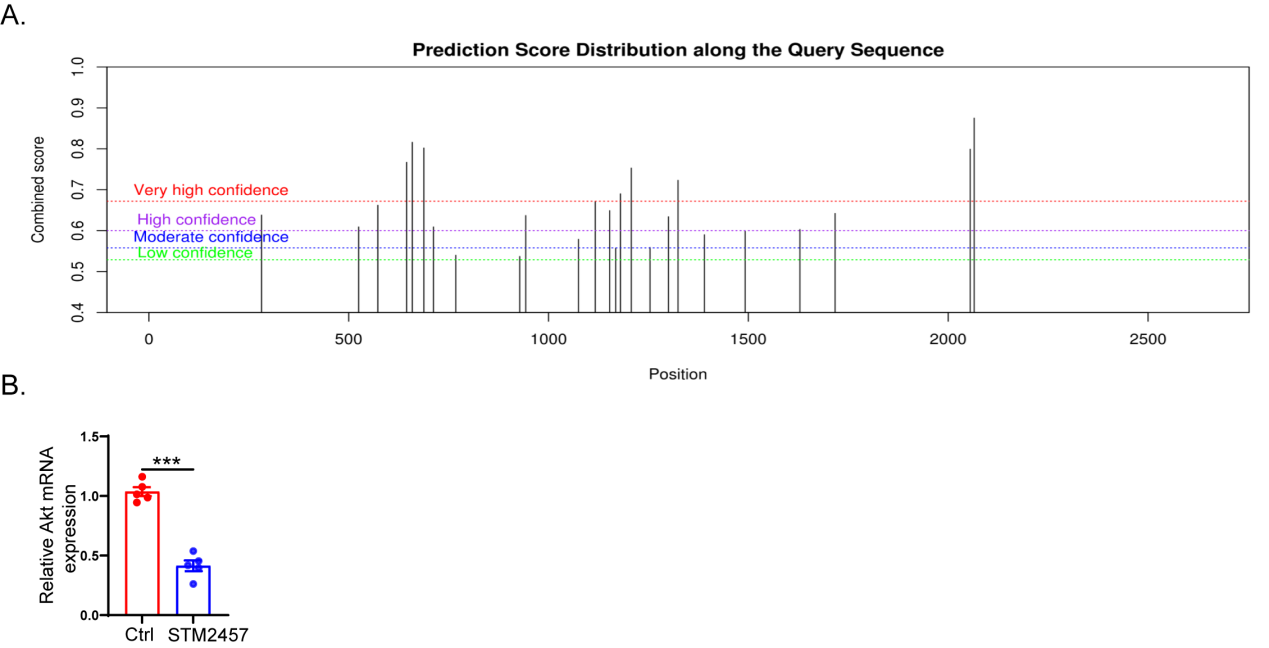


**Supplementary Figure 10:** METTL3 is involved in catalyzing Akt m^6^A modification. (A) Predicted binding sites of m^6^A modification at Akt sequence according to the online SRAMP database (<http://www.cuilab.cn/sramp>). (B) The m^6^A level of Akt mRNA in CFs treated with STM2457 (5 μM) (n = 5). Data are expressed as mean ± SEM. Results were analyzed using one-way ANOVA with Tukey's *post hoc* test and confirmed by three independent experiments. ****p* < 0.001 vs. Ctrl.

Supplementary Figure 11:


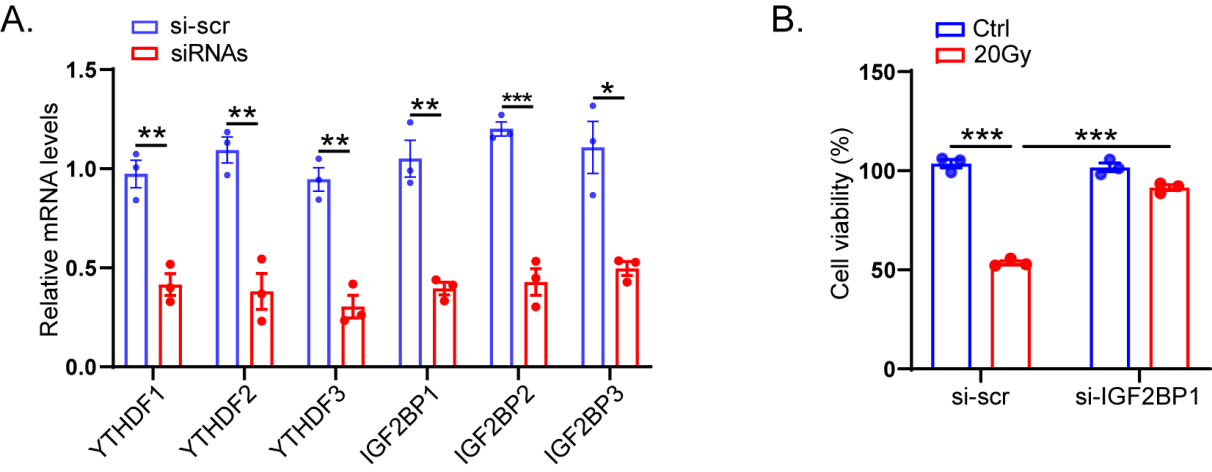


**Supplementary Figure 11:** Knockdown of IGF2BP1 inhibits radiation-induced decrease in cell activity. (A) RT-PCR analysis showing the knockdown of readers in CFs (n = 5). (B)Cell viability of CFs irradiated with or without IGF2BP1 knockdown(n = 5).

Supplementary Figure 12:


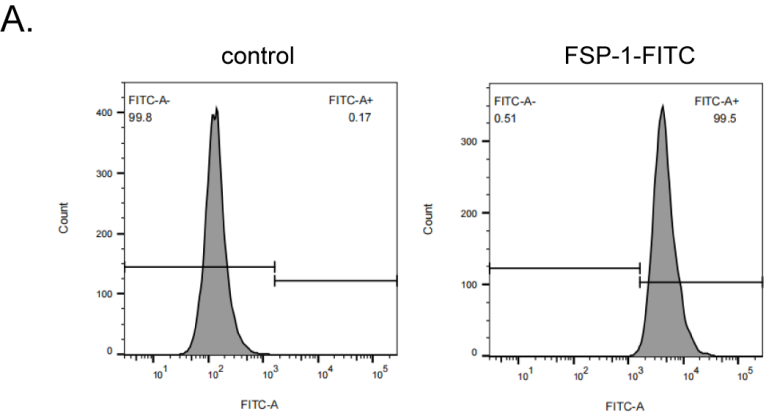


**Supplemental Figure S12:** Cardiac fibroblasts identification. Isolated CF was identified by flow cytometry.
