# Supplementary figures and images for: METTL3 Modulates Radiation‐Induced Cardiac Fibrosis via the Akt/mTOR Pathway
Source: FASEB J. 2025 Jun 5;39(11):e70666. doi: 10.1096/fj.202403143RRRR (PMC12139579; doi:10.1096/fj.202403143RRRR)

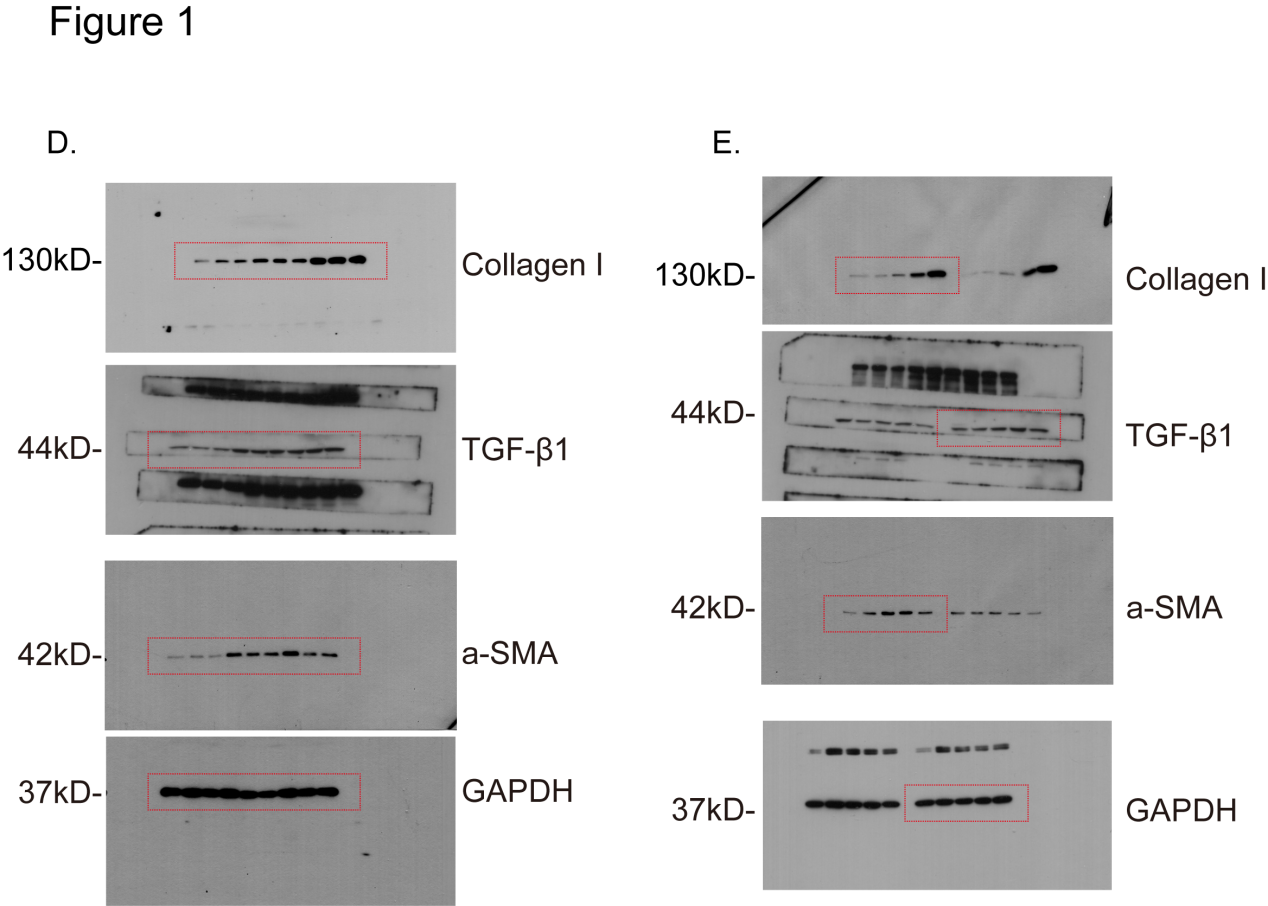


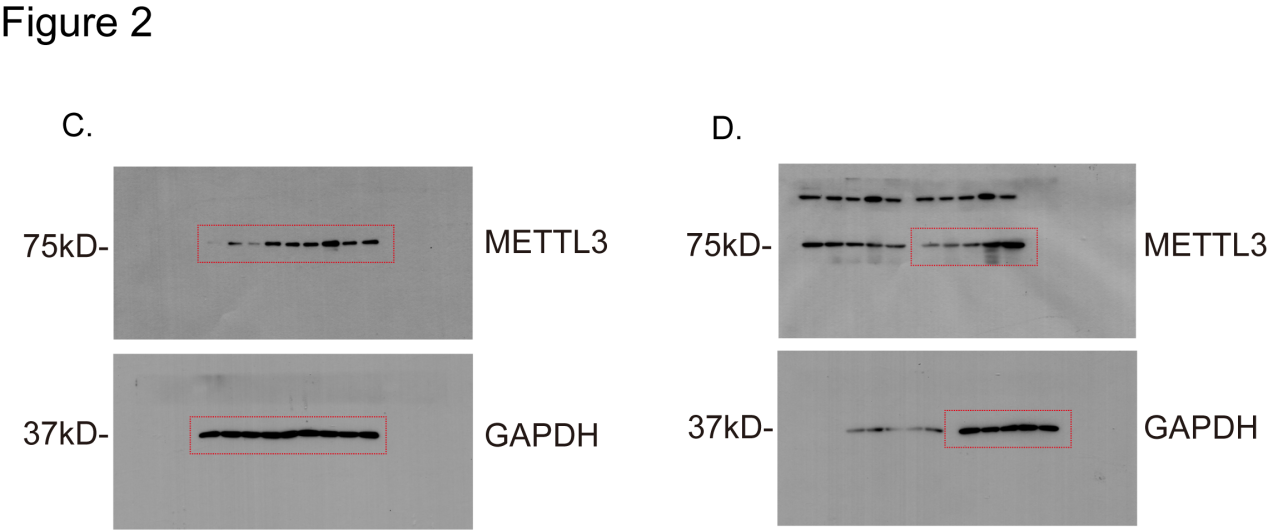


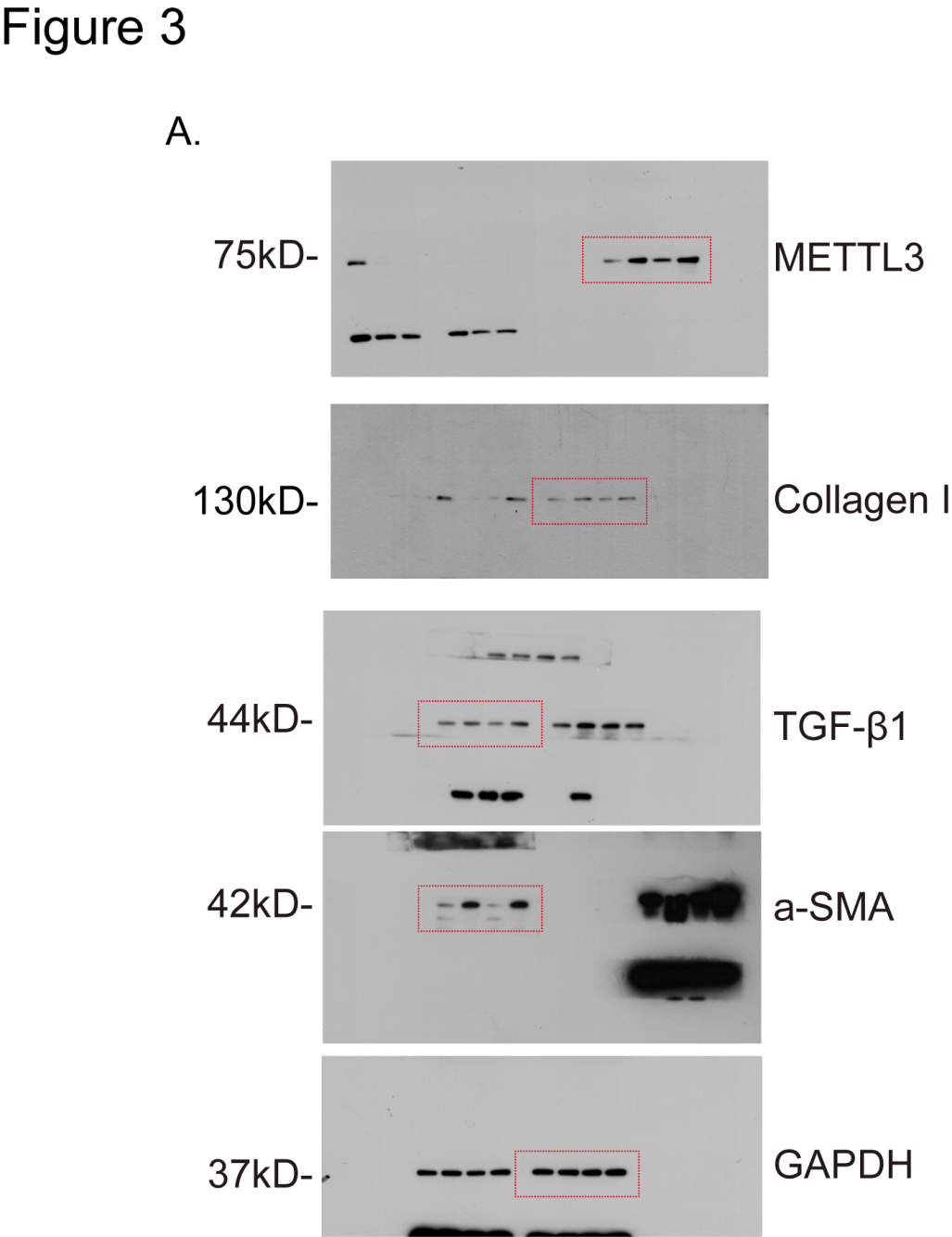


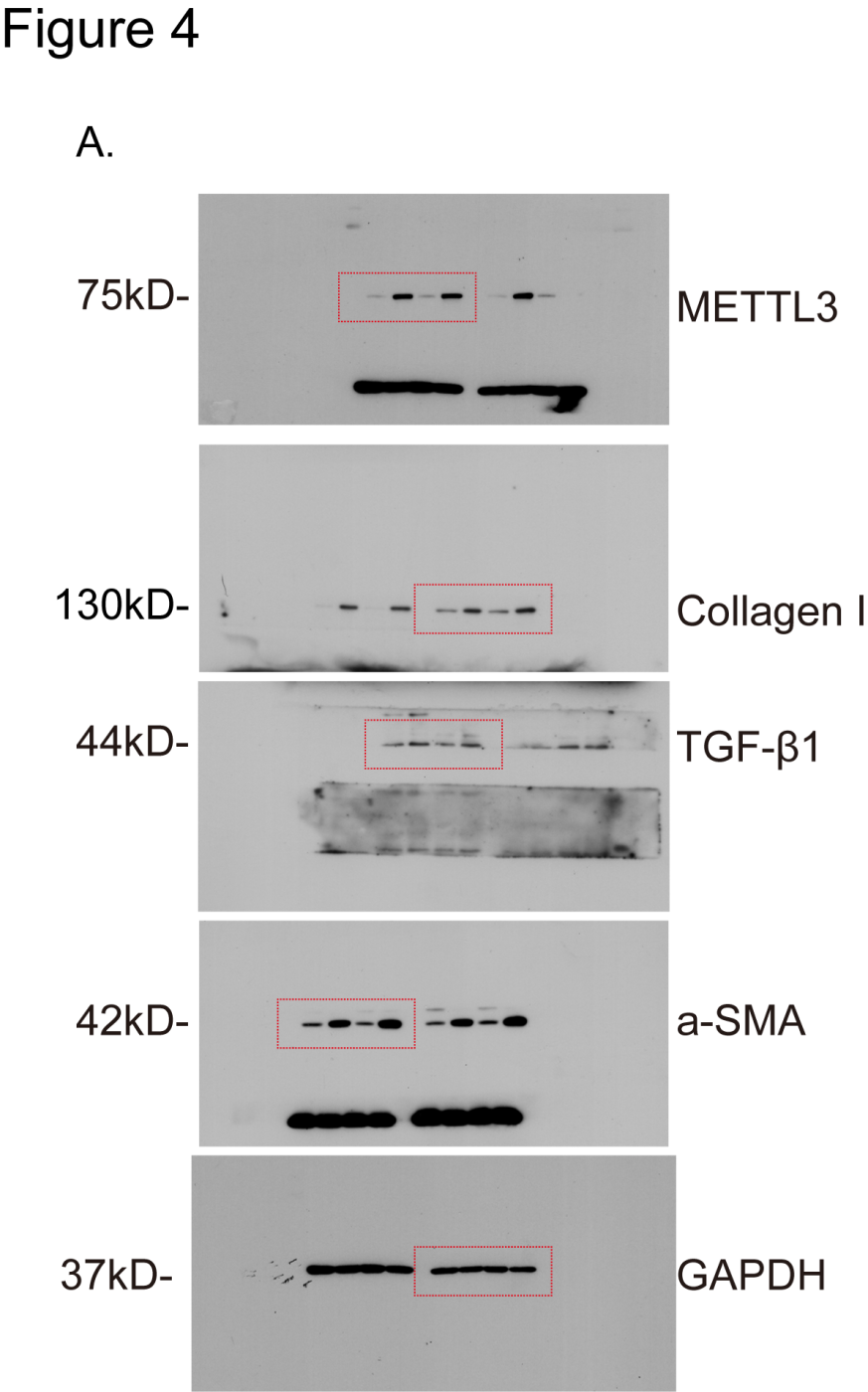


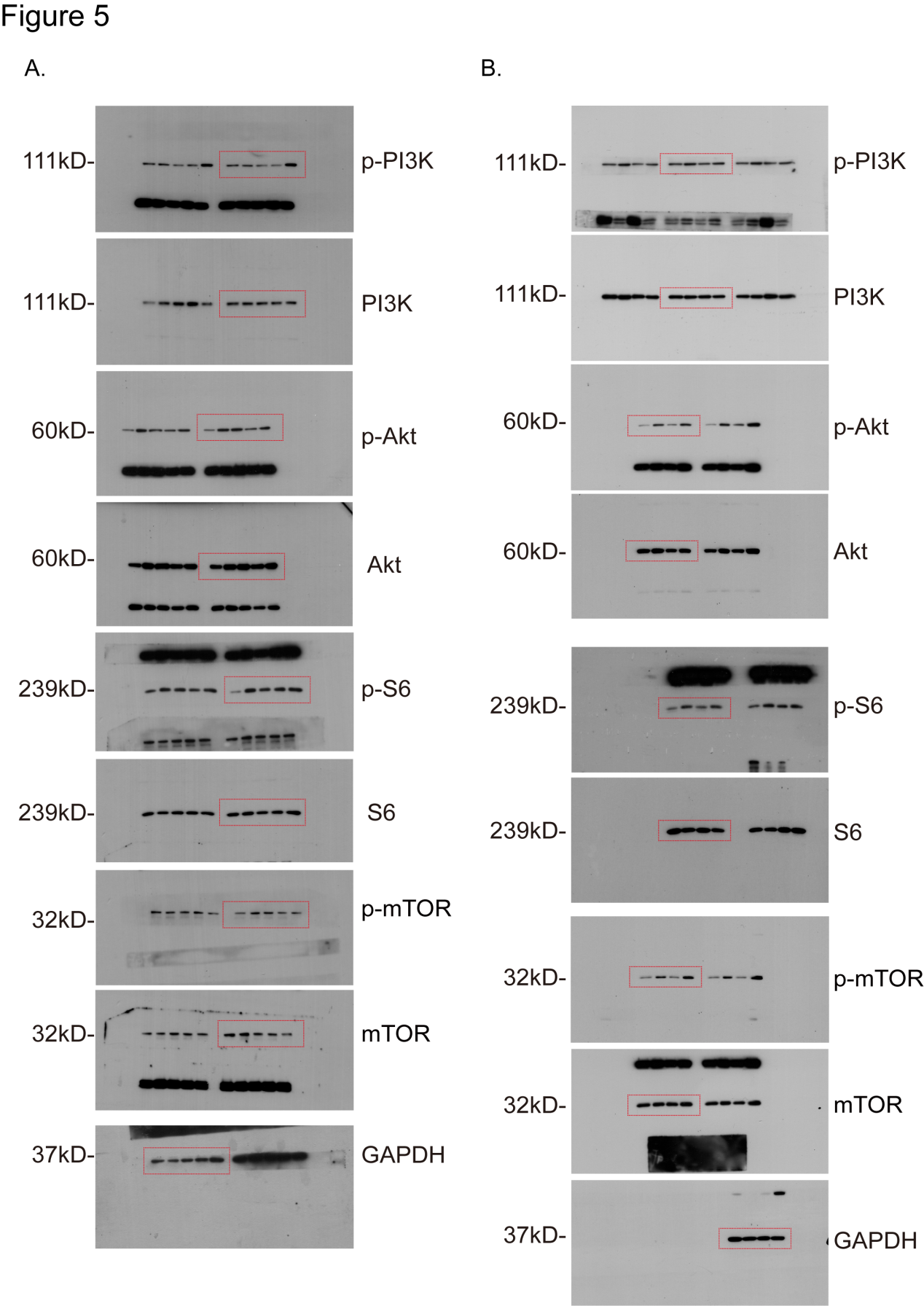


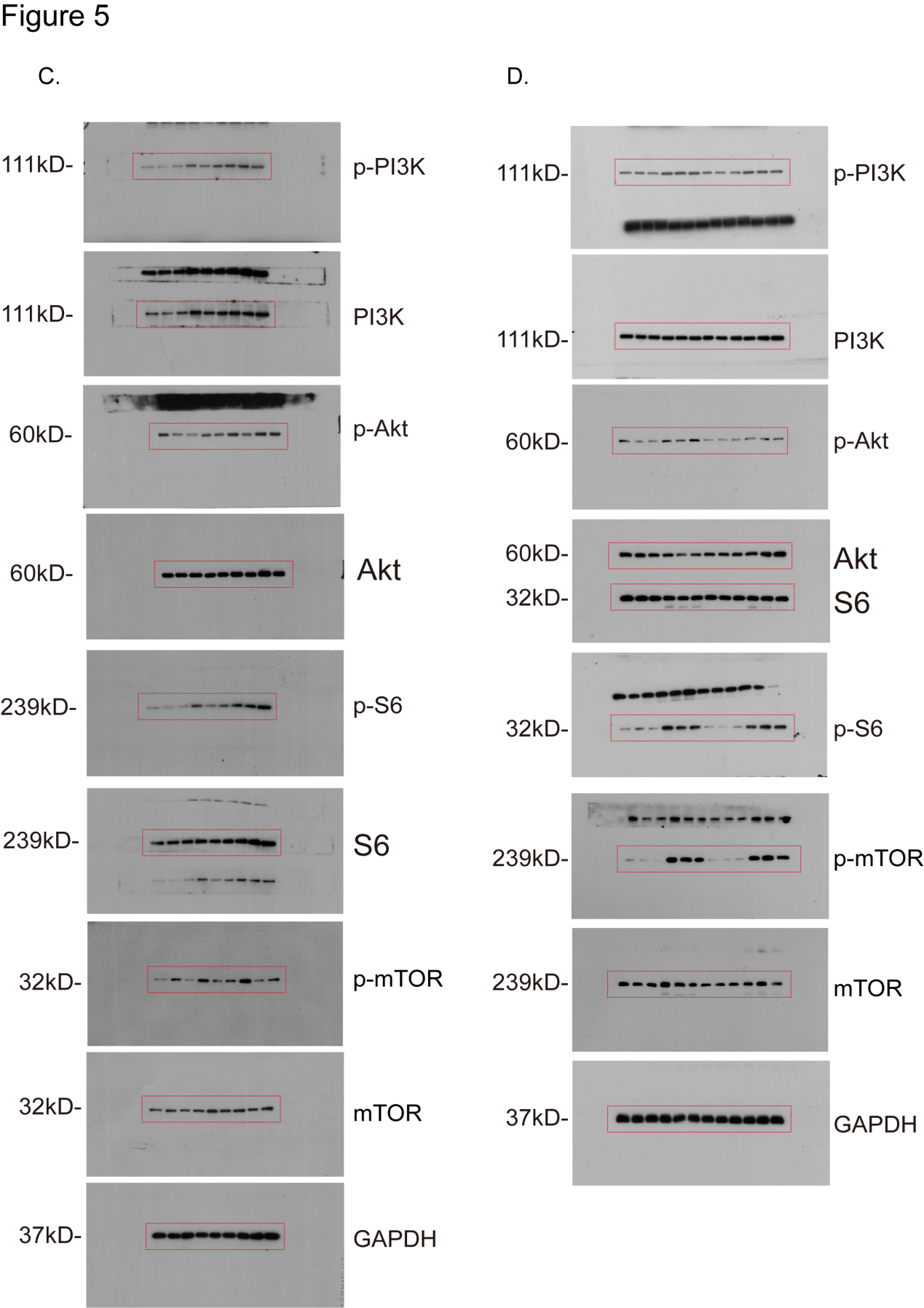


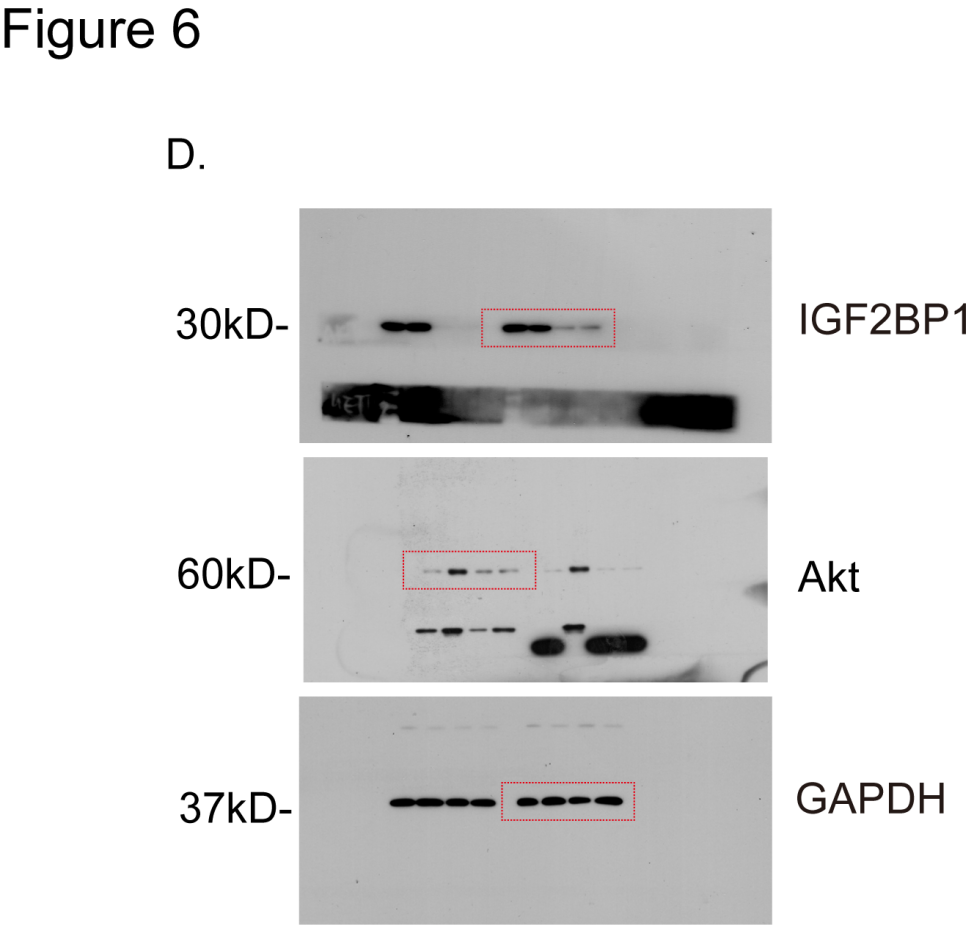


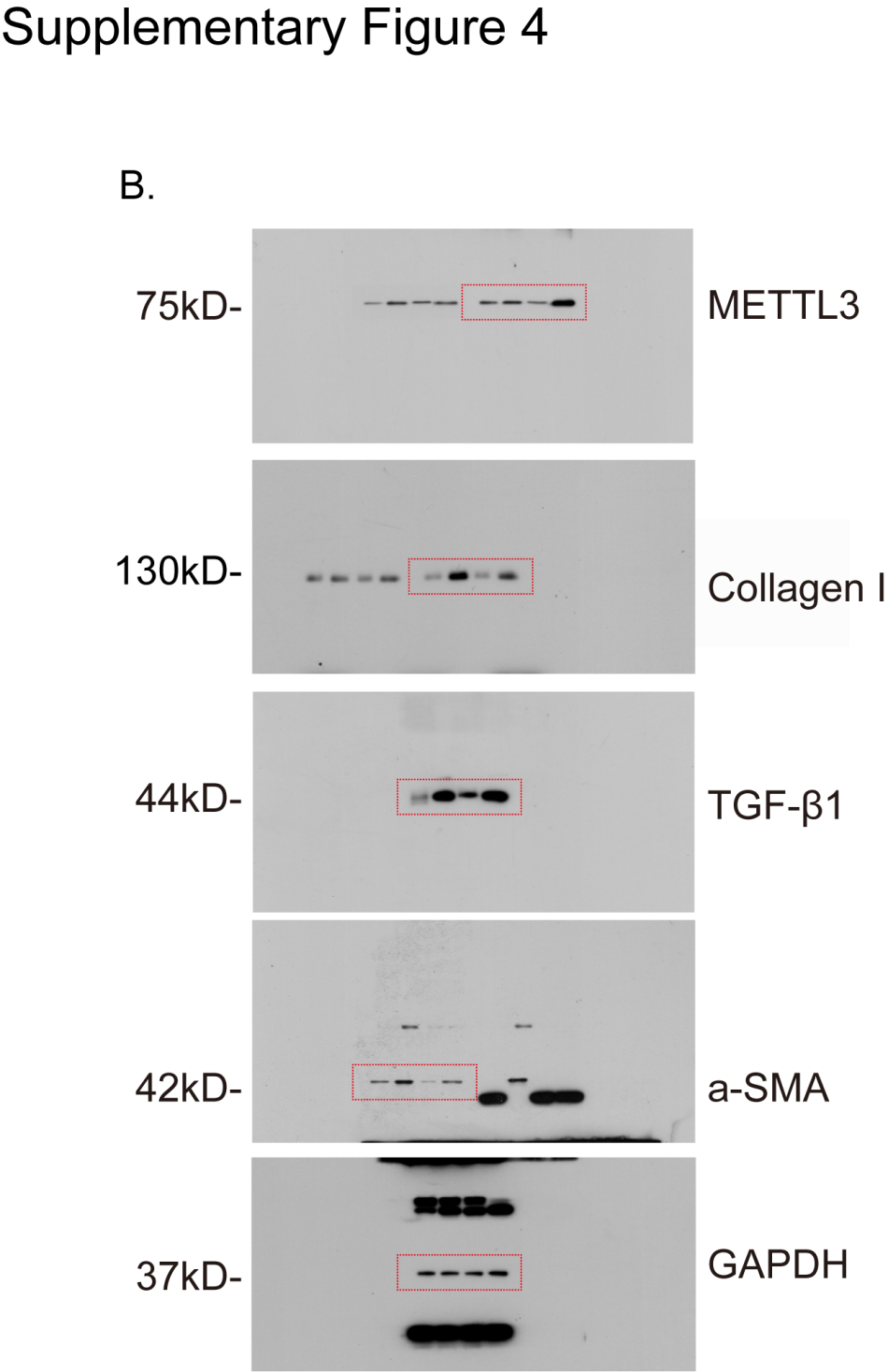


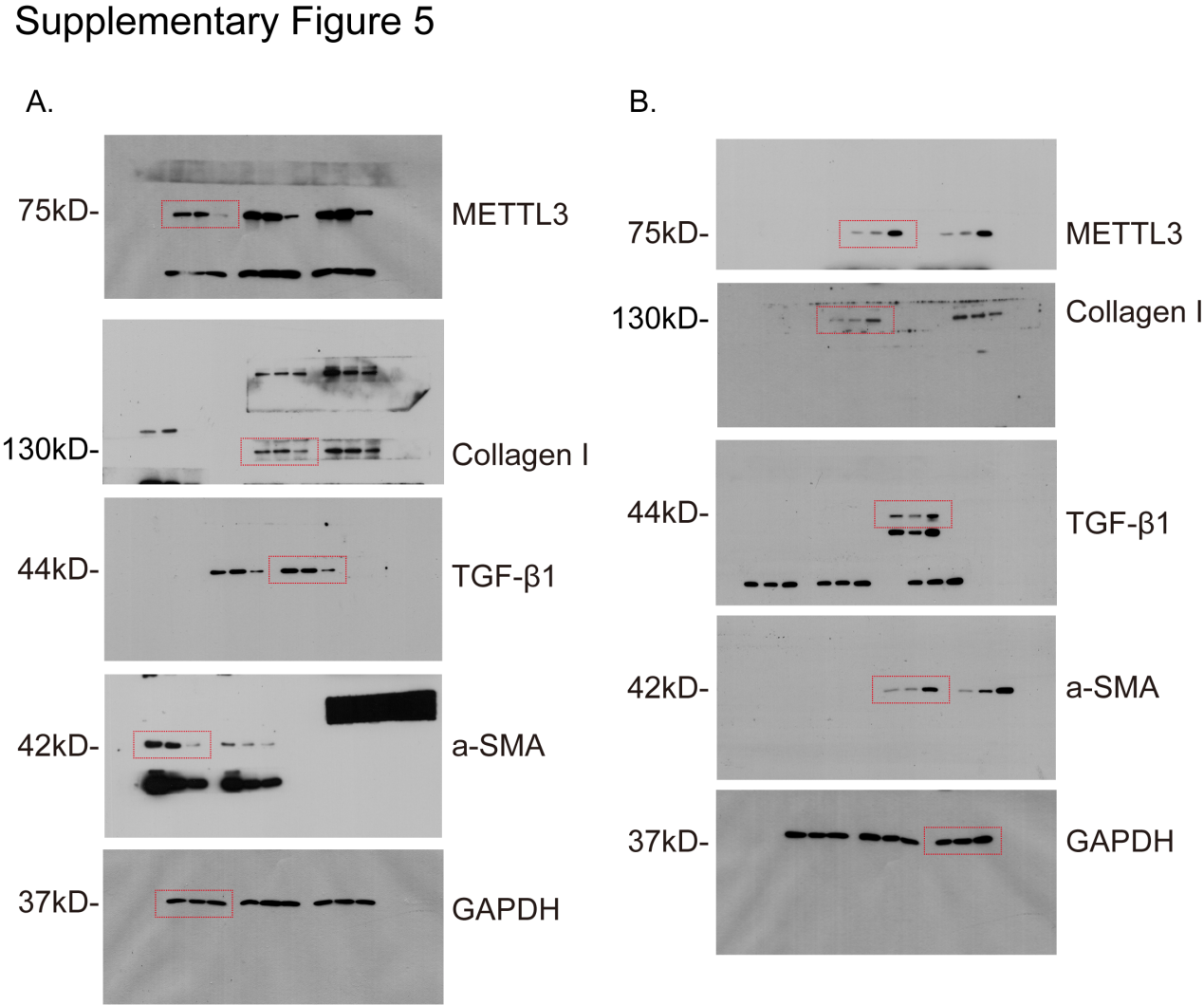


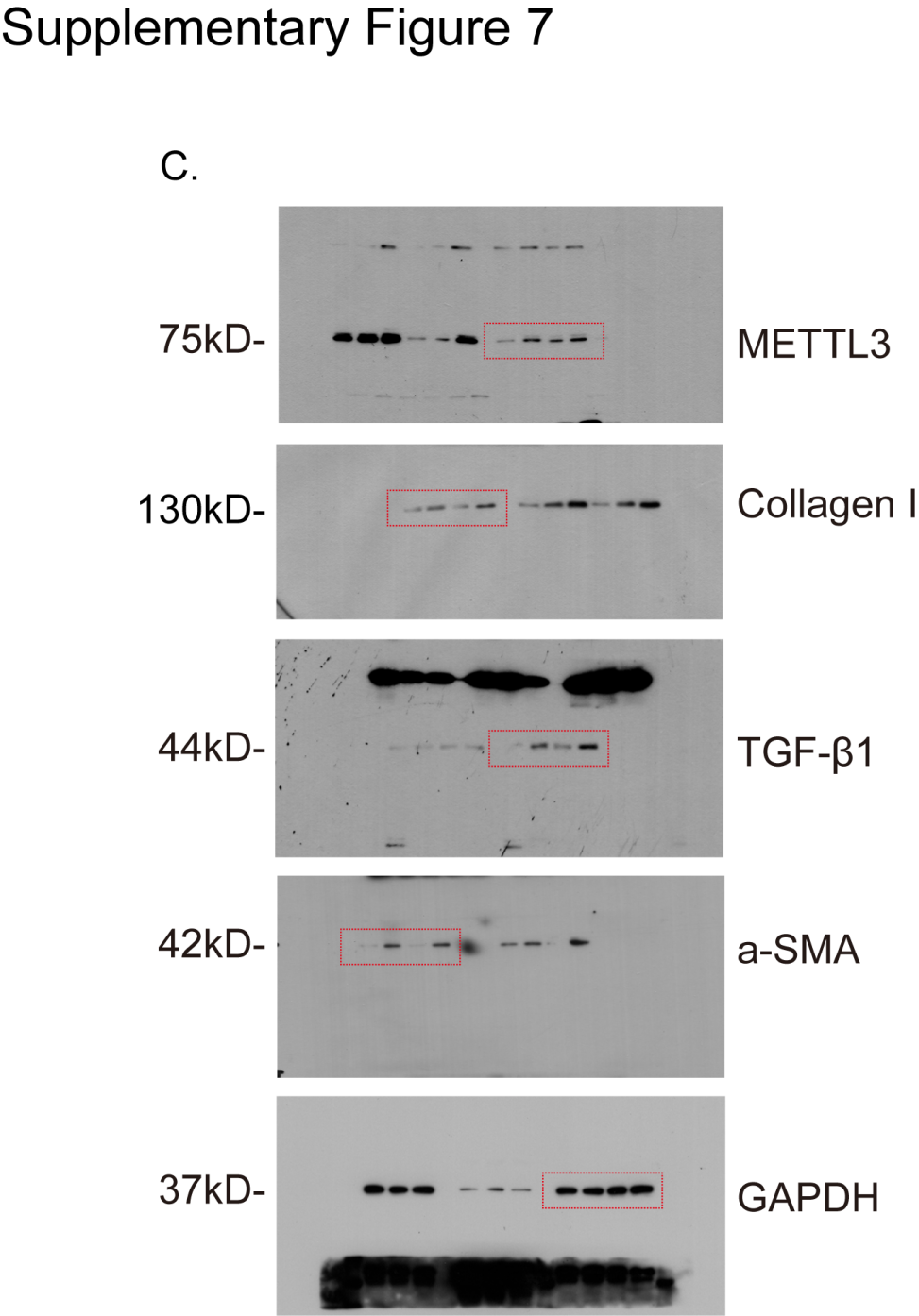


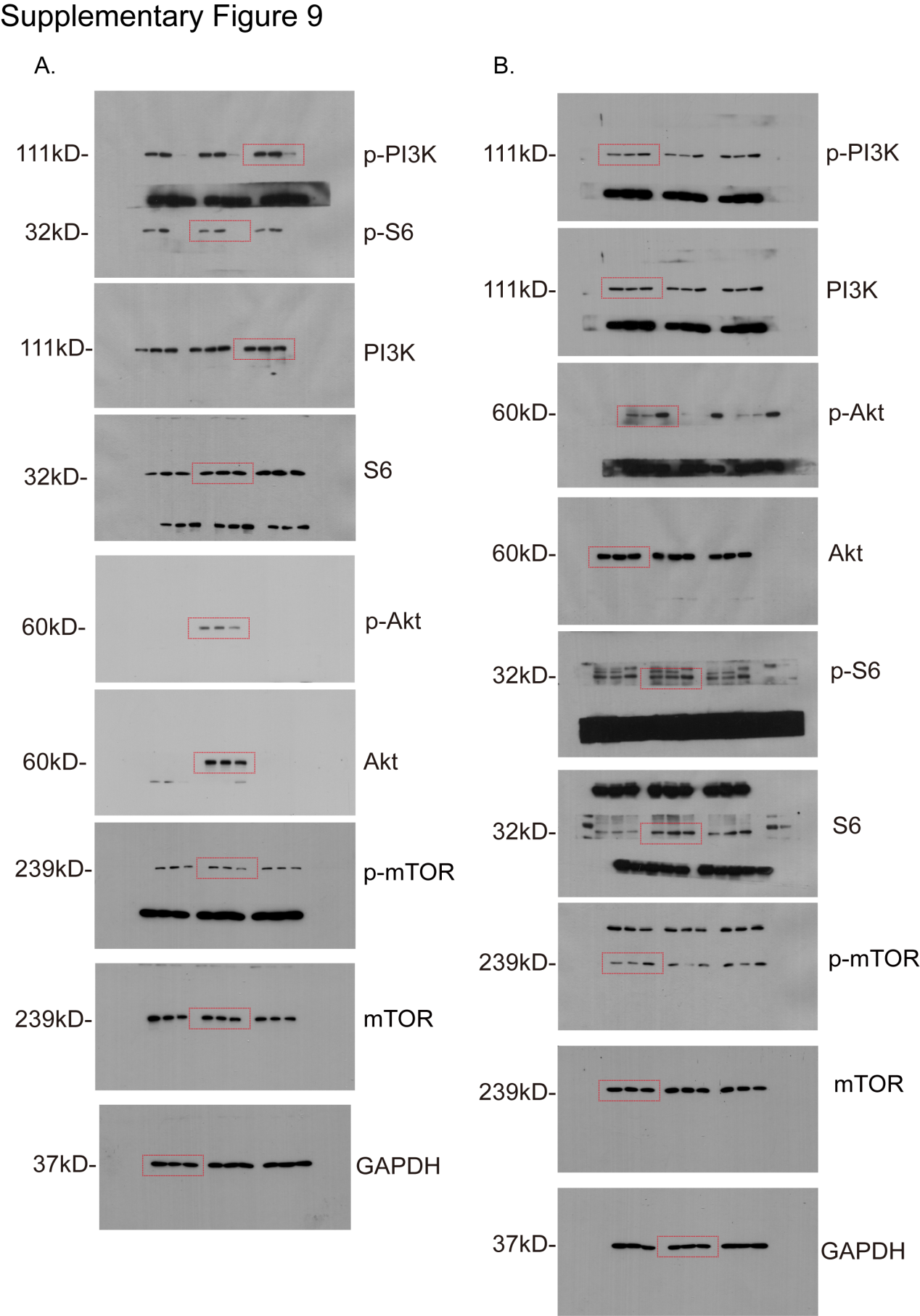

Supplement: Supplementary file 3 — Data S2. [file FSB2-39-e70666-s002.docx]
