# Supplementary material for: METTL3 Modulates Radiation‐Induced Cardiac Fibrosis via the Akt/mTOR Pathway
Source: FASEB J. 2025 Jun 5;39(11):e70666. doi: 10.1096/fj.202403143RRRR (PMC12139579; doi:10.1096/fj.202403143RRRR)
Supplement: Supplementary file 4 — Table S4. [file FSB2-39-e70666-s001.docx]

**Table S4**. Primer sequences used in this study

| **Gene** | **Sequence** | **Application** |
| --- | --- | --- |
| Akt | F: 5’-ATGAACGACGTAGCCATTGTG-3’  R: 5’-TTGTAGCCAATAAAGGTGCCAT-3’ | RT-PCR |
| YTHDF1 | F: 5’-ACAGTTACCCCTCGATGAGTG-3’  R: 5’-GGTAGTGAGATACGGGATGGGA-3’ | RT-PCR |
| YTHDF2 | F: 5’-GAGCAGAGACCAAAAGGTCAAG-3’  R: 5’-CTGTGGGCTCAAGTAAGGTTC-3’ | RT-PCR |
| YTHDF3 | F: 5’-CATAGGGCAACAGAGGAAACAG-3’  R: 5’-ATCTCCAGCCGTGGACCAT-3’ | RT-PCR |
| IGF2BP1 | F: 5’-CAAATGGGTGACTATGGACTGAC-3’  R: 5’-TGAGGACTGAGGTAGTTCATCC-3’ | RT-PCR |
| IGF2BP2 | F: 5’-GTCCTACTCAAGTCCGGCTAC-3’  R: 5’-CATATTCAGCCAACAGCCCAT-3’ | RT-PCR |
| IGF2BP3 | F: 5’-CCTGGTGAAGACGGGCTAC-3’  R: 5’-TCAACTTCCATCGGTTTCCCA-3’ | RT-PCR |
| GAPDH | F: 5’-AGGTCGGTGTGAACGGATTTG-3’  R: 5’-TGTAGACCATGTAGTTGAGGTCA-3’ | RT-PCR |
| Akt Site 1 | F: 5’-CUCGGAGUAGGAGCAGGAAG-3’  R: 5’-UGGCCGAGCGGAGGCCUGGG-3’ | MeRIP-qPCR |
| Akt Site 2 | F: 5’-UAAAACCUGGCGGCCACGCU-3’  R: 5’-ACUUCCUCCUCAAGAACGAU-3’ | MeRIP-qPCR |
| Akt Site 3 | F: 5’-GGCACCUUUAUUGGCUACAA-3’  R: 5’-AUCAGCGAGAGUCCCCACUC-3’ | MeRIP-qPCR |
| Akt Site 4 | F: 5’-AACAACUUCUCAGUGGCACA-3’  R: 5’-AUGCCAGCUGAUGAAGACAG-3’ | MeRIP-qPCR |
| Akt Site 5 | F: 5’-AGCGGCCAAGGCCCAACACC-3’  R: 5’-UUUAUCAUCCGCUGCCUGCA-3’ | MeRIP-qPCR |
| Akt Site 6 | F: 5’-UUGAGCGCACCUUCCAUGUG-3’  R: 5’-GAAACGCCUGAGGAGCGGGA-3’ | MeRIP-qPCR |
| Akt Site 7 | F: 5’-AGAAUGGGCCACCGCCAUUC-3’  R: 5’-AGACUGUGGCAGAUGGACUC-3’ | MeRIP-qPCR |
| Akt Site 8 | F: 5’-AAGAGGCAGGAAGAAGAGAC-3’  R: 5’-GAUGGACUUCCGAUCAGGCU-3’ | MeRIP-qPCR |

**Table S5**. The predicted 25 m^6^A residues in Akt mRNA sequence predicted with the SRAMP database (http://www.cuilab.cn/sramp)

| **#** | **Position** | **Sequence context** | **Score** | **Decision** |
| --- | --- | --- | --- | --- |
| 1 | 282 | GGGCUCAGCCUACCGAGAAGAGACUCUGAGCAUCAUCCCUGGGUU | 0.638 | (High confidence) |
| 2 | 525 | GGCACAAUGCCAGCUGAUGAAGACAGAGCGGCCAAGGCCCAACAC | 0.609 | (High confidence) |
| 3 | 573 | UAUCAUCCGCUGCCUGCAGUGGACCACAGUCAUUGAGCGCACCUU | 0.662 | (High confidence) |
| 4 | 645 | AGAAUGGGCCACCGCCAUUCGGACU  GUGGCAGAUGGACUCAAGAG | 0.767 | (Very high confidence) |
| 5 | 659 | CCAUUCAGACUGUGGCAGAUGGACUCAAGAGGCAGGAAGAAGAGA | 0.816 | (Very high confidence) |
| 6 | 688 | AGGCAGGAAGAAGAGACGAUGGACUUCCGAUCAGGCUCACCCAGU | 0.802 | (Very high confidence) |
| 7 | 712 | UUCCGAUCAGGCUCACCCAGUGACAACUCAGGGGCUGAAGAGAUG | 0.609 | (High confidence) |
| 8 | 768 | GGCCAAGCCCAAGCACCGUGUGACCAUGAACGAGUUUGAGUACCU | 0.54 | (Low confidence) |
| 9 | 928 | GUUGCCCACACGCUUACUGAGAACCGUGUCCUGCAGAACUCUAGG | 0.537 | (Low confidence) |
| 10 | 943 | ACUGAGAACCGUGUCCUGCAGAACUCUAGGCAUCCCUUCCUUACG | 0.637 | (High confidence) |
| 11 | 1075 | CGAGAGCGUGUGUUCUCCGAGGACCGGGCCCGCUUCUAUGGUGCG | 0.579 | (Moderate confidence) |
| 12 | 1117 | GCGGAGAUUGUGUCUGCCCUGGACUACUUGCACUCCGAGAAGAAC | 0.671 | (High confidence) |
| 13 | 1153 | GAGAAGAACGUGGUGUACCGGGACCUGAAGCUGGAGAACCUCAUG | 0.649 | (High confidence) |
| 14 | 1168 | UACCGGGACCUGAAGCUGGAGAACCUCAUGCUGGACAAGGACGGG | 0.557 | (Low confidence) |
| 15 | 1180 | AAGCUGGAGAACCUCAUGCUGGACUAGGACGGGCACAUCAAGAUA | 0.69 | (Very high confidence) |
| 16 | 1207 | GACGGGCACAUCAAGAUAACGGACUUCGGGCUGUGCAAGGAGGGG | 0.753 | (Very high confidence) |
| 17 | 1254 | CAAGGACGGUGCCACUAUGAAGACAUUCUGCGGAACGCCGGAGUA | 0.559 | (Moderate confidence) |
| 18 | 1300 | CUGGCCCCUGAGGUGCUGGAGGACAACGACUACGGCCGUGCAGUG | 0.634 | (High confidence) |
| 19 | 1324 | AACGACUACGGCCGUGCAGUGGACUGGUGGGGGCUGGGCGUGGUC | 0.723 | (Very high confidence) |
| 20 | 1390 | CGCCUGCCCUUCUACAACCAGGACCACGAGAAGCUGUUCGAGCUG | 0.59 | (Moderate confidence) |
| 21 | 1492 | CUCUCCGGGCUGCUCAAGAAGGACCCUACACAGAGGCUCGGUGGG | 0.599 | (Moderate confidence) |
| 22 | 1629 | CAAGCCCCAGGUCACCUCUGAGACUGACACCAGGUAUUUCGAUGA | 0.603 | (High confidence) |
| 23 | 1717 | GAUGACAGCAUGGAGUGUGUGGACAGUGAGCGGAGGCCGCACUUC | 0.642 | (High confidence) |
| 24 | 2055 | AACAAUUAGAUUCAUGUAGAGGACUAUUAAGGACUGACGCGACCA | 0.799 | (Very high confidence) |
| 25 | 2065 | UUCAUGUAGAAAACUAUUAAGGACUGACGCGACCAUGUGCAAUGU | 0.875 | (Very high confidence) |
